# Cytoskeletal remodeling promotes tunneling nanotube formation and drives cardiac resident cell mitochondrial transfer in sepsis

**DOI:** 10.1126/sciadv.adz3266

**Published:** 2026-03-11

**Authors:** Rui Song, Cheng Huang, Yinrui Ma, Zhenhua Zhang, Yifei Liu, Bing Chen, Xi Zhang, Shuai Hao, He Huang, Milad Ashrafizadeh, João Conde, Chenyang Duan

**Affiliations:** ^1^ Department of Critical Care Medicine and Anesthesiology, The Second Affiliated Hospital of Chongqing Medical University, Chongqing 400010, P.R. China.; ^2^ The First Affiliated Hospital of Hunan University of Medicine, Huihua 418000, P.R. China.; ^3^ School of Pharmaceutical Sciences, Sun Yat-sen University, Guangzhou 510006, P.R. China.; ^4^ The First College of Clinical Medical Science, China Three Gorges University, Yichang 443008, P.R. China.; ^5^ Research Institute of General Surgery, Jinling Hospital, Affiliated Hospital of Medical School, Nanjing University, Nanjing 210002, P.R. China.; ^6^ Department of Radiation Oncology, Shandong Provincial Key Laboratory of Radiation Oncology, Shandong Cancer Hospital and Institute, Shandong First Medical University, Shandong Academy of Medical Sciences, Jinan 250000, P.R. China.; ^7^ Comprehensive Health Research Centre (CHRC), NOVA Medical School, Faculdade de Ciências Médicas, NMS|FCM, Universidade NOVA de Lisboa, Lisboa, Portugal.

## Abstract

Sepsis-induced cardiac dysfunction arises from complex intercellular communication networks that extend beyond direct cardiomyocyte damage, yet the nanoscale mechanisms governing these interactions remain poorly understood. Here, we identify tunneling nanotubes (TNTs) as dynamic biological nanostructures facilitating intercellular mitochondrial transfer, revealing their critical role in septic cardiac remodeling. Using a murine cecal ligation and puncture (CLP) model and single-cell RNA sequencing, we demonstrate that sepsis reprograms cardiac endothelial cells, fibroblasts, and macrophages, generating metabolically impaired subpopulations with dysfunctional mitochondrial respiration. We uncover a Drp1-driven cytoskeletal remodeling process that orchestrates TNT biogenesis, wherein Drp1 interacts with Filamin and Kinesin to regulate TNT formation and extension, enabling long-range organelle trafficking. Cardiac-specific Drp1 knockout disrupts TNT-mediated mitochondrial exchange, halting metabolic deterioration and reversing cellular reprogramming. These findings establish Drp1-mediated TNT networks as nanoscale conduits of organelle communication, offering insights into biological nanotube engineering, cellular-scale nanotechnology, and potential therapeutic interventions for mitochondrial dysfunction in sepsis.

## Teaser

Cytoskeletal remodeling by Drp1 promotes nanotube formation and mitochondrial transfer in septic hearts.

## INTRODUCTION

Sepsis is a life-threatening condition characterized by a dysregulated systemic inflammatory response to infection, often culminating in multiple organ dysfunction and high mortality rates. It remains a primary cause of death in critically ill perioperative patients (*1*). Globally, sepsis affects approximately 49 million individuals annually and accounts for an estimated 11 million deaths, representing ~20% of global fatalities. These statistics highlight the considerable burden on public health and urgent need for effective therapeutic strategies (*2*). Among the complications of sepsis, cardiac dysfunction occurs in ~40% of patients, characterized by reduced ventricular contractility and diminished ejection fraction. This condition considerably increases mortality risk, imposing a profound burden on families and health care systems (*3*). Understanding the mechanisms underlying sepsis-induced cardiac dysfunction and identifying previously unidentified therapeutic targets are crucial to improve clinical outcomes.

While much research on sepsis-induced cardiac dysfunction has focused on cardiomyocyte damage (*4*) and impaired function (*5*), emerging evidence suggests that noncardiomyocyte resident cardiac cells, such as endothelial cells, fibroblasts, and macrophages, also play vital roles in maintaining cardiac homeostasis (*6*). Endothelial cells regulate vascular permeability and coagulation, maintain structural stability, mediate repair, and contribute to immune responses by clearing harmful substances and mediating inflammation. During sepsis, these cells undergo notable functional alterations that disrupt intercellular communication and mitochondrial dynamics, thereby exacerbating cardiac dysfunction. For example, endothelial cell activation during early sepsis dysregulates extracellular signal–regulated kinase/mitogen-activated protein kinase signaling, shifting endothelial cells from a proliferative to a proapoptotic phenotype. This transition disrupts tight junctions, leading to increased microvascular permeability and coagulation dysfunction, which are key drivers of hypotension, heart failure, and arrhythmia (*7*). Similarly, fibroblast activation triggers NOD-like receptor family pyrin domain containing 3 (NLRP3) inflammasome activation, promoting interleukin-1β (IL-1β) release and impairing sarcoplasmic/endoplasmic reticulum (ER) Ca^2+^–adenosine triphosphatase 2a expression in cardiomyocytes, thereby contributing to contractile dysfunction (*8*). Moreover, macrophages amplify inflammatory cascades through the overproduction of cytokines such as tumor necrosis factor–α, IL-6, IL-8, and IL-1β, further impairing cardiomyocyte function and driving systemic inflammation (*9*). These findings emphasize the need to explore the role of noncardiomyocytes and their contribution to sepsis-induced cardiac dysfunction.

Intercellular communication, particularly mitochondrial transfer, is critical for the maintenance of cellular homeostasis under stressful conditions. Mitochondrial transfer is crucial in energy supply, metabolic regulation, cell repair, and immune modulation (*10*). Cells achieve mitochondrial transfer through several mechanisms, including tunneling nanotubes (TNTs), gap junctions, extracellular vesicles, and free mitochondrial capture (*11*). Among these, TNTs stand out for their ability to transport not only small molecules such as cytokines and microRNAs but also large organelles such as intact mitochondria over long distances. This unique capability makes TNT-mediated mitochondrial transfer highly relevant in various pathological and physiological contexts (*12*). For instance, TNT-mediated mitochondrial transfer enhances mitochondrial respiration in CD8^+^ T cells, overcomes exhaustion in T cell immunotherapy (*13*), and mitigates neuronal damage in neurodegenerative diseases by transferring healthy mitochondria to the compromised neurons (*14*). However, the role of TNT-mediated mitochondrial communication in sepsis-induced cardiac dysfunction remains unclear.

Dynamin-related protein 1 (Drp1), a key regulator of mitochondrial fission, is implicated in sepsis (*15*). In addition to its role in mitochondrial dynamics, Drp1 interacts with cytoskeletal proteins such as Filamin and Kinesin, influencing cytoskeletal remodeling (*16*). Given the importance of cytoskeletal dynamics in TNT formation, Drp1 has been hypothesized to facilitate TNT-mediated mitochondrial transfer during sepsis. However, the role of this mechanism in sepsis-induced cardiac dysfunction remains unclear.

In the present study, we investigated the role of Drp1-mediated cytoskeletal remodeling in TNT formation and mitochondrial transfer during sepsis. Using a cecal ligation and puncture (CLP) mouse model to simulate sepsis, we combined single-cell transcriptomics and high-resolution imaging to analyze functional transitions in cardiac resident cells. We reveal that sepsis induces considerable functional alterations in cardiac endothelial cells, fibroblasts, and macrophages, with newly emerging subpopulations exhibiting mitochondrial dysfunction and metabolic abnormalities. In addition, Drp1 undergoes conformational changes during sepsis, mediating cytoskeletal remodeling through interactions with Filamin and Kinesin, which, in turn, facilitate TNT formation and extension. This study offers insights into the interplay between mitochondrial dynamics, intercellular communication, and cardiac dysfunction in sepsis and provides potential therapeutic targets for mitigating organ damage.

## RESULTS

### Cardiac dysfunction and cellular transcriptomic alterations during sepsis progression

**Fig. 1.**
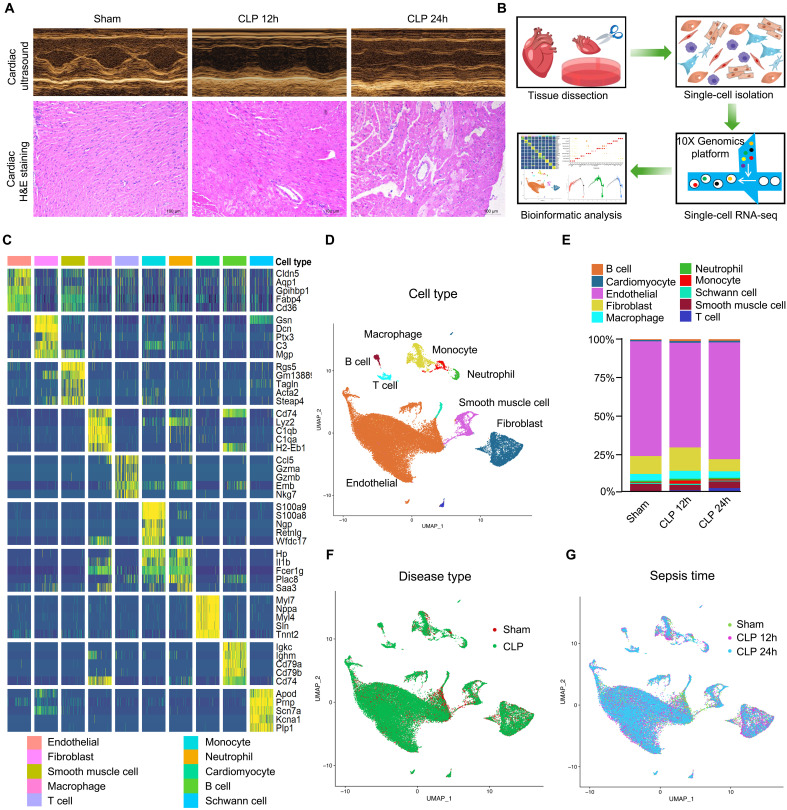
scRNA-seq characterization of heart tissues from septic mice. (**A**) Ultrasound and hematoxylin and eosin (H&E)–stained images of heart tissues from Sham and sepsis group mice; *n* = 8 per group. Scale bars, 100 μm. (**B**) Schematic diagram of mouse heart tissue processing and scRNA-seq process. (**C**) Heatmap of the expression of five marker genes defining the 10 major cell clusters. (**D**) UMAP of the integrated transcriptome from the Sham, CLP 12h, and CLP 24h groups displaying the distribution of 10 major cell clusters. (**E**) Histogram of the integrated transcriptome from the Sham, CLP 12h, and CLP 24h groups showing the proportion of 10 major cell clusters. (**F**) UMAP of the integrated transcriptome of the Sham, CLP 12h, and CLP 24h groups showing cell distribution in the disease and normal groups. (**G**) UMAP of the integrated transcriptome from the Sham, CLP 12h, and CLP 24h groups showing cell distribution at different time points. CLP, cecal ligation puncture; UMAP, uniform manifold approximation and projection.

To investigate the effects of sepsis on cardiac function and pathology, we established a severe sepsis model in C57BL/6 mice using CLP, mimicking abdominal infection. Compared with control mice, 12-hour (12h) post-CLP mice displayed markedly reduced cardiac contractility, with irregular myocardial fiber arrangement, interstitial edema, and hemorrhage. By 24 hours post-CLP, both diastolic and systolic cardiac functions had further deteriorated, consistent with progression to septic shock. Pathological examination of the myocardial tissues showed exacerbated histological changes, including the appearance of pale pink degenerative cells, suggesting a potential link between cardiac dysfunction and different types of cellular degeneration during sepsis (Fig. 1A).

To delineate the gene expression landscape of cardiac tissues at single-cell resolution across different stages of sepsis, we performed 10× single-cell RNA sequencing (scRNA-seq) of heart samples collected from the Sham, CLP 12h, and CLP 24h groups (*n* = 2 per group; Fig. 1B). After quality control, transcriptomic data were obtained from 44,289 single cells. Cell populations were identified using cluster marker analysis, manual annotation, and validation against external reference signatures, yielding a comprehensive atlas of cell types (Fig. 1C; fig. S1, A and B; and table S1).

We identified 10 major cell types, endothelial cells (*n* = 32,537), fibroblasts (*n* = 5117), smooth muscle cells (*n* = 1907), macrophages (*n* = 2047), T cells (*n* = 587), monocytes (*n* = 482), neutrophils (*n* = 458), cardiomyocytes (*n* = 449), B cells (*n* = 416), and Schwann cells (*n* = 289) (Fig. 1, D and E). Uniform manifold approximation and projection (UMAP)–based cluster visualization revealed significant temporal changes in the expression patterns of cluster-defining markers in cardiac tissues as sepsis progressed (Fig. 1, F and G).

### Sepsis-induced transformation of cardiac endothelial cell subpopulations

**Fig. 2.**
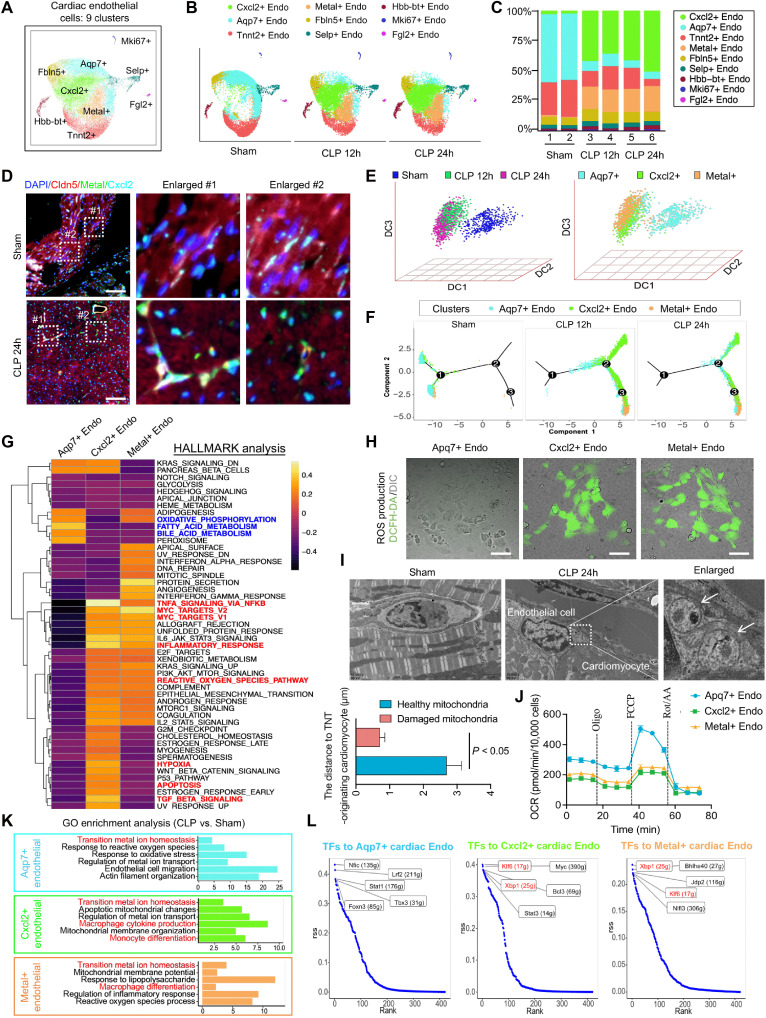
Clustering and functional analysis of cardiac endothelial cell subclusters using scRNA-seq. (**A**) UMAP of nine subclusters of cardiac endothelial cells in the Sham, CLP 12h, and CLP 24h groups with integrated transcriptomes. (**B**) Distribution of nine subclusters of cardiac endothelial cells with prolonged duration after sepsis UMAP. (**C**) Histogram of the proportion of nine subclusters of cardiac endothelial cells in each group. (**D**) mIHC staining images showing the expression and distribution of 4′,6-diamidino-2-phenylindole (DAPI), Cldn5, Metal, and Cxcl2 in cardiac endothelial cells after sepsis; *n* = 8 per group. Scale bars, 25 μm. (**E**) Analysis of Aqp7+, Cxcl2+, and Metal+ cardiac endothelial cells (right) and their diffusion components (DCs) with prolonged sepsis duration (left). (**F**) Pseudotime trajectory analysis of Aqp7+, Cxcl2+, and Metal+ cardiac endothelial cells with significant changes after sepsis at different time points. (**G**) HALLMARK functional analysis heatmap of Aqp7+, Cxcl2+, and Metal+ cardiac endothelial cells with significant changes after sepsis. (**H**) ROS detection of Aqp7+, Cxcl2+, and Metal+ cardiac endothelial cells; *n* = 3 per group. Scale bars, 50 μm. (**I**) TEM images showing the morphology of cardiac endothelial cells in mice of the Sham or sepsis group; *n* = 8 per group. Scale bars, 2 μm. (**J**) Mitochondrial respiration assay of Aqp7+, Cxcl2+, and Metal+ cardiac endothelial cells; *n* = 3 per group. (**K**) GO enrichment analysis of Aqp7+, Cxcl2+, and Metal+ cardiac endothelial cells after sepsis. (**L**) Top five TFs with the largest differences in expression regulatory estimated in the cardiac endothelial cell subclusters of Aqp7+ Cxcl2+, and Metal+ after sepsis. scRNA-seq, single-cell RNA sequencing; ROS, reactive oxygen species; TEM, transmission electron microscopy; GO, Gene Ontology; TF transcription factor; OCR, oxygen consumption rate; Endo, endothelial cells; rss, regulon specificity score.

The 32,537 identified cardiac endothelial cells were grouped into nine distinct clusters based on the scRNA-seq results (Fig. 2, A and B, and fig. S2A). Marker gene analysis revealed that during sepsis, the Aqp7+ endothelial subpopulation was progressively replaced by two novel subpopulations, Cxcl2+ and Metal+ endothelial cells (Fig. 2C). Using multiplex immunohistochemistry (mIHC), we further validated the distribution and abundance of Cxcl2+ and Metal+ endothelial subpopulations in the cardiac tissues of CLP mice and observed a significant increase in the number of Cxcl2-Cldn5 and Metal-Cldn5 double-positive cells, consistent with clustering analysis results (Fig. 2D and fig. S2B). Pseudotime trajectory analysis provided a clearer depiction of the transition from Aqp7+ to Cxcl2+ and Metal+ endothelial subpopulations during sepsis progression (Fig. 2, E and F, and fig. S2, C and D).

To elucidate the biological significance of this transition, HALLMARK pathway analysis was performed, which revealed that the declining Aqp7+ subpopulation represented mitochondrial oxidative metabolism–active endothelial cells. In contrast, the increased Cxcl2+ and Metal+ subpopulations exhibited characteristics of mitochondrial oxidative stress and cellular damage, including reactive oxygen species (ROS) accumulation, excessive inflammation, hypoxia-induced apoptosis, and impaired mitochondrial function (Fig. 2G and fig. S2E). DCFH-DA (2′,7′-dichlorodihydrofluorescein diacetate) detection analysis revealed significantly elevated intracellular ROS levels in the Cxcl2+ and Metal+ endothelial subpopulations compared with those in Aqp7+ endothelial cells (Fig. 2H and fig. S2F). Transmission electron microscopy (TEM) further revealed the accumulation of damaged mitochondria in endothelial cells adjacent to cardiomyocytes in CLP mice (Fig. 2I). Seahorse-based metabolic assays demonstrated that mitochondrial respiratory capacity was significantly higher in the Aqp7+ subpopulation than in the Cxcl2+ and Metal+ subpopulations, as indicated by their lower basal and maximal respiration rates (Fig. 2J and fig. S2, G and H). These findings indicated that sepsis induces the accumulation of damaged mitochondria in a subset of resident cardiac endothelial cells. The healthy Aqp7+ endothelial subpopulation, characterized by active mitochondrial metabolism, gradually disappears, whereas the inflammatory and metabolically deficient Cxcl2+ and Metal+ subpopulations become predominant. In addition, Gene Ontology (GO) enrichment analysis linked endothelial cell transition to the changes in mitochondrial quality, metal ion transport, and monocyte/macrophage differentiation (Fig. 2K and fig. S2I).

Last, we investigated the transcription factors (TFs) driving the transition of cardiac endothelial cells from the Aqp7+ cluster to the Cxcl2+ and Metal+ clusters, using single-cell regulatory network inference and clustering (SCENIC) (fig. S3A). SCENIC identified Nfic, Irf2, Stat1, Tbx3, and Foxn3; Klf6, Myc, Xbp1, Bcl3, and Stat3; Xbp1, Bhlhe40, Jdp2, Klf6, and Nlfi3 as the top candidate TFs for Aqp7+, Cxcl2+, and Metal+ endothelial cells, respectively (Fig. 2L and fig. S3B). Trajectory analysis of TFs and their target genes revealed that the down-regulation of TFs such as Nfic may accelerate the loss of Aqp7+ endothelial cells, while the up-regulation of TFs such as Klf6 and Xbp1 may promote the emergence of Cxcl2+ and Metal+ endothelial subpopulations (fig. S3, C to F).

### Sepsis-induced transformation of cardiac fibroblast cell subpopulations

**Fig. 3.**
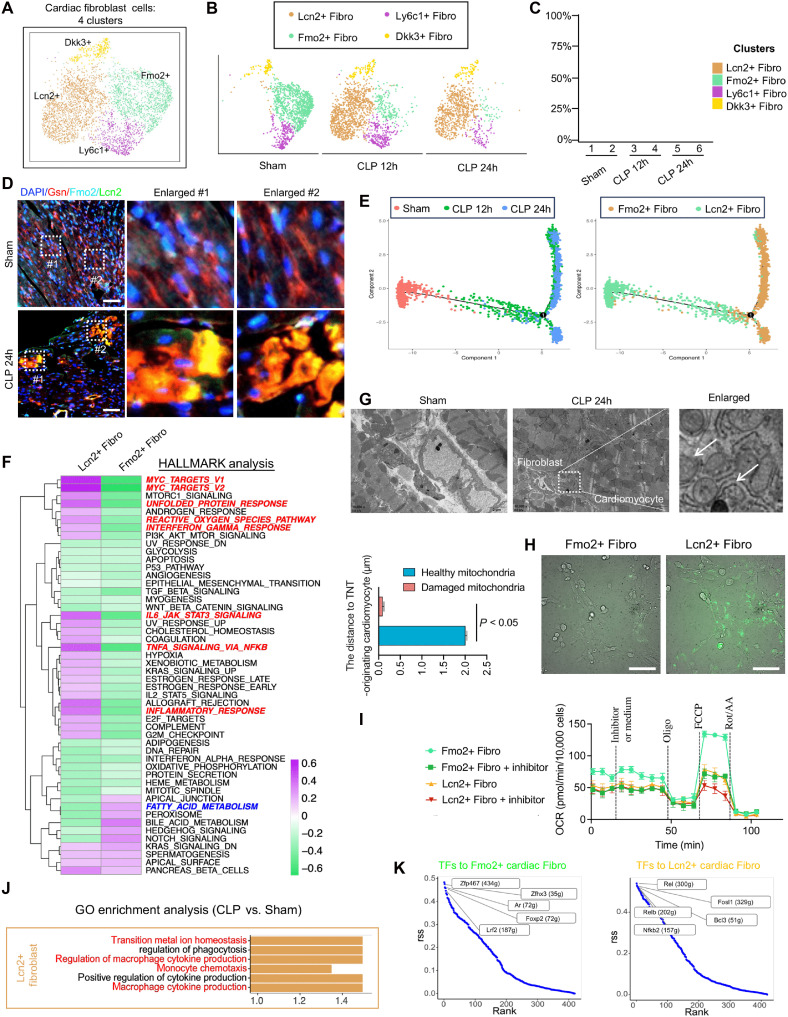
Clustering and functional analysis of CF subclusters using scRNA-seq. (**A**) UMAP of four subclusters of CFs in the Sham, CLP 12h, and CLP 24h groups with integrated transcriptomes. (**B**) Distribution of four subclusters of CFs with prolonged duration after sepsis UMAP. (**C**) Histogram of the proportion of four subclusters of CFs in each group. (**D**) mIHC staining images showing the expression and distribution of DAPI, Gsn, Fmo2, and Lcn2 in CFs after sepsis; *n* = 8 per group. Scale bars, 25 μm. (**E**) Pseudotime trajectory analysis of Fmo2+ and Lcn2+ CF subclusters (right) with significant changes after sepsis and their changes with sepsis duration (left). (**F**) HALLMARK functional analysis heatmap of Lcn2+ and Fmo2+ CFs with significant changes after sepsis. (**G**) TEM images showing the morphology of CFs in mice of the Sham or sepsis groups; *n* = 8 per group. Scale bars, 2 μm. (**H**) ROS detection of Fmo2+ and Lcn2+ CFs; *n* = 3 per group. Scale bars, 50 μm. (**I**) Mitochondrial fatty acid metabolism capacity assay of Fmo2+ and Lcn2+ CFs after a Seahorse XF24 assay and flow cytometry; *n* = 3 per group. (**J**) GO enrichment analysis of Lcn2+ CFs after sepsis. (**K**) Top five TFs with the largest differences in expression estimated in the postsepsis Fmo2+ and Lcn2+ CFs. Fibro, fibroblasts.

The 5117 cardiac fibroblast (CF) cells identified were grouped into four distinct clusters, Lcn2+, Fmo2+, Ly6c1+, and Dkk3+ fibroblast cells (Fig. 3A). Marker genes for each cluster were identified (fig. S4A). Trajectory analysis revealed a notable transition in CFs during sepsis, with the Fmo2+ subpopulation gradually disappearing and the Lcn2+ subpopulation emerging (Fig. 3, B and C). Using mIHC, we assessed the abundance and distribution of Fmo2+ and Lcn2+ fibroblast subpopulations in the hearts of Sham and CLP 24h mice. Fmo2-Gsn double-positive fibroblast levels were significantly reduced, whereas Lcn2-Gsn double-positive fibroblast levels were markedly increased in CLP 24h mice compared with those in the Sham group, consistent with clustering analysis results (Fig. 3D and fig. S4B). Pseudotime trajectory analysis further depicted the transition from Fmo2+ to Lcn2+ fibroblast subpopulations during sepsis progression (Fig. 3E).

HALLMARK pathway analysis revealed that each fibroblast subpopulation relied on distinct metabolic pathways for energy production, Ly6c1+ fibroblasts primarily used oxidative phosphorylation, Dkk3+ fibroblasts relied on glycolysis, and Fmo2+ fibroblasts depended on fatty acid metabolism (Fig. 3F and fig. S4C). The loss of Fmo2+ fibroblasts during sepsis likely contributes to impaired cardiac fatty acid metabolism, whereas Lcn2+ fibroblast emergence may exacerbate oxidative stress and inflammatory damage via MYC-mediated immune evasion and excessive inflammatory activation. TEM revealed the accumulation of damaged mitochondria in fibroblasts near cardiomyocytes in CLP mice (Fig. 3G). DCFH-DA detection analysis showed that ROS levels were significantly higher in Lcn2+ than in Fmo2+ fibroblasts (Fig. 3H and fig. S4D). Seahorse analysis of fatty acid metabolism demonstrated that the maximal oxygen consumption rate response of Fmo2+ fibroblasts was approximately three times higher than that of Lcn2+ fibroblasts (Fig. 3I and fig. S4, E and F), indicating that the newly emerging Lcn2+ fibroblasts exhibited reduced fatty acid metabolism, consistent with HALLMARK analysis results. GO enrichment analysis further associated the excessive inflammatory response in Lcn2+ fibroblasts during sepsis with processes such as metal ion transport, macrophage cytokine production, and monocyte chemotaxis (Fig. 3J and fig. S4G).

Last, SCENIC analysis identified key TFs regulating fibroblast transition. The down-regulation of TFs such as Zfp467, Zfhx3, Ar, Foxp2, and Lrf2 may accelerate the loss of Fmo2+ fibroblasts, whereas the up-regulation of TFs such as Rel, Fosl1, Relb, Bcl3, and Nf-kb2 may promote the emergence of Lcn2+ fibroblast subpopulations during sepsis progression (Fig. 3K and fig. S4H).

### Sepsis-induced transformation of cardiac monocyte and macrophage subpopulations

**Fig. 4.**
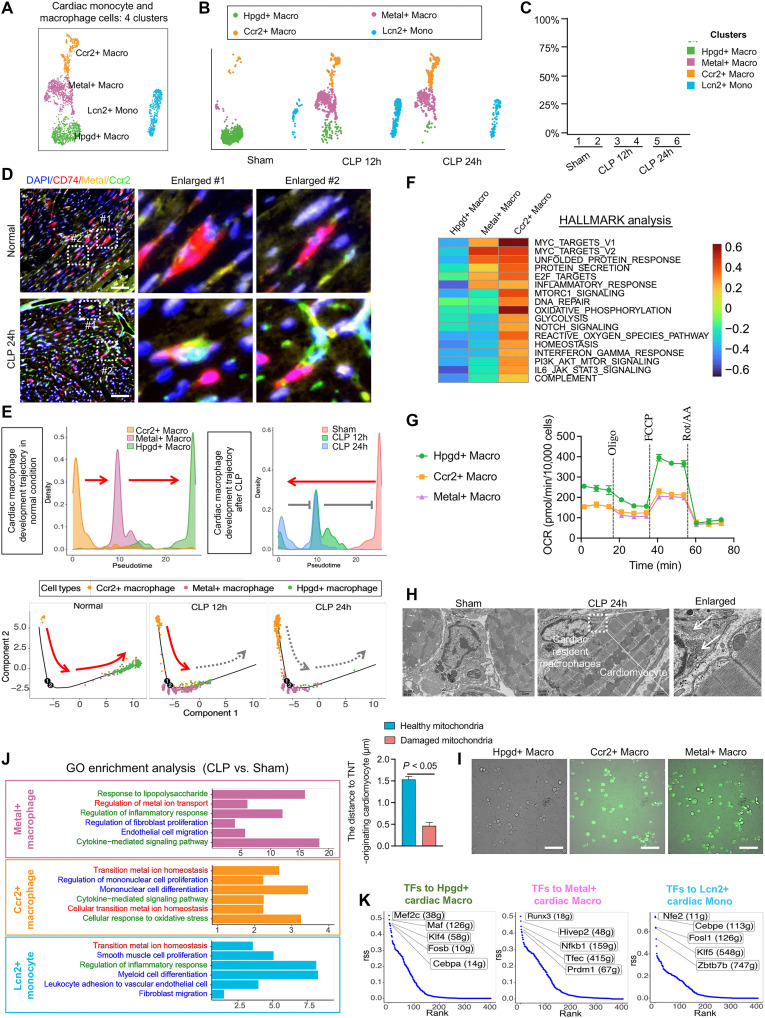
Clustering and functional analysis of cardiac monocyte-macrophage subclusters using scRNA-seq. (**A**) UMAP of four subclusters of cardiac monocytes-macrophages in the Sham, CLP 12h, and CLP 24h group integrated transcriptomes. (**B**) UMAP distribution of four subclusters of cardiac monocytes-macrophages with the duration of sepsis. (**C**) Histogram of the proportion of four subclusters of cardiac monocytes-macrophages in each group. (**D**) mIHC staining images showing the expression and distribution of DAPI, CD74, Metal, and Ccr2 in cardiac monocytes-macrophages after sepsis; *n* = 8 per group. Scale bars, 25 μm. (**E**) Pseudotime trajectory of the occurrence and development of Ccr2+, Metal+, and Hpgd+ cardiac macrophage subclusters under normal conditions (left); pseudotime trajectory of the occurrence and development of cardiac macrophages with the prolongation of sepsis duration (right); and pseudotime trajectory of Ccr2+, Metal+, and Hpgd+ cardiac macrophage subclusters with the duration of sepsis (bottom). (**F**) HALLMARK functional analysis heatmap of Hpgd+, Metal+, and Ccr2+ cardiac macrophages with significant changes after sepsis. (**G**) Mitochondrial respiration assay of Hpgd+, Metal+, and Ccr2+ cardiac macrophages; *n* = 3 per group. (**H**) TEM images showing the morphology of cardiac macrophages in mice of the Sham or sepsis group; *n* = 8 per group. Scale bars, 2 μm. (**I**) ROS detection of Hpgd+, Metal+, and Ccr2+ cardiac macrophages; *n* = 3 per group. Scale bars, 50 μm. (**J**) GO enrichment analysis of Metal+ and Ccr2+ cardiac macrophages after sepsis. (**K**) Top five TFs with the largest differences in expression estimated in the postsepsis Hpgd+ and Metal+ cardiac macrophages and Lcn2+ cardiac monocytes. Macro, macrophages; Mono, monocytes.

To investigate the link between the emergence of new endothelial and fibroblast subpopulations and macrophage differentiation or cytokine production during sepsis, we analyzed the changes in cardiac monocyte and macrophage populations. A total of 2496 cardiac monocytes and macrophages were identified and grouped into four clusters, Hpgd+, Metal+, and Ccr2+ macrophages and Lcn2+ monocytes (Fig. 4A). Marker genes for each cluster were identified (fig. S5A). Trajectory analysis revealed a transition in the cardiac monocyte and macrophage populations during sepsis, with the Hpgd+ macrophage subpopulation gradually disappearing and Lcn2+ monocytes and Metal+ macrophages emerging (Fig. 4, B and C, and fig. S5B). Consistent with these clustering results, mIHC showed that Metal+ and Ccr2+ macrophage levels were significantly increased in the cardiac tissues of CLP 24h mice compared with those in Sham controls (Fig. 4D and fig. S5C). Pseudotime trajectory analysis further depicted the transition from Hpgd+ macrophages to Metal+ macrophages during sepsis progression (Fig. 4E).

HALLMARK pathway analysis revealed that the shift from Hpgd+ to Metal+ macrophages reflected a reduction in fatty acid metabolism and oxidative phosphorylation capacity, accompanied by enhanced proinflammatory polarization, mitochondrial unfolded protein response damage, and MYC-mediated immune evasion activation (Fig. 4F). In addition, significantly increased counts of Ccr2+ macrophages were associated with elevated proinflammatory responses, mitochondrial damage, reduced fatty acid metabolism and oxidative phosphorylation, enhanced glycolytic capacity, and MYC-mediated immune evasion inactivation (Fig. 4F). Seahorse metabolic assays demonstrated that the basal and maximal respiration rates of Ccr2+ and Metal+ macrophages were significantly lower than those of Hpgd+ macrophages, indicating impaired mitochondrial respiratory capacity in these emergent subpopulations, consistent with HALLMARK analysis results (Fig. 4G and fig. S5, D and E). TEM revealed the accumulation of damaged mitochondria in macrophages near cardiomyocytes in CLP 24h mice (Fig. 4H). DCFH-DA detection confirmed the significantly higher ROS levels in Ccr2+ and Metal+ macrophages than in Hpgd+ macrophages (Fig. 4I and fig. S5F). GO enrichment analysis showed that Hpgd+ macrophages were closely related to monocyte migration and inflammation, whereas emerging Metal+ macrophages were associated with metal ion transport and endothelial cell changes. This suggests a potential link between the Metal+ endothelial and Metal+ macrophage subpopulations during sepsis. Similarly, the significantly increased Lcn2+ monocytes were closely associated with metal ion transport (Fig. 4J).

Last, SCENIC analysis identified the TFs that regulate these transitions. The down-regulation of TFs such as Mef2c, Maf, Klf4, Fosb, and Cebpa may accelerate the disappearance of Hpgd+ macrophages. In contrast, the up-regulation of TFs such as Runx3, Hivep2, Nf-kb1, Tfec, and Prdm1 may promote the emergence of Metal+ macrophages, while the up-regulation of Nfe2, Cebpe, Fosl1, Klf5, and Zbtb7b may drive the emergence of Lcn2+ monocytes (Fig. 4K and fig. S5G). Notably, a retrospective analysis of SCENIC data revealed that Fosl1 plays a role in the transitions of both Lcn2+ cardiac monocytes and Lcn2+ CFs during sepsis.

### Drp1-mediated TNT formation facilitates intercellular mitochondrial transfer and dysfunction in cardiac resident cells during sepsis

Our findings revealed that most cardiac resident cells, including endothelial cells, fibroblasts, and macrophages, accumulated damaged mitochondria after sepsis, predominantly near cardiomyocytes. Since the mitochondrial content in noncardiomyocyte resident cells is insufficient to explain the emergence of damaged mitochondria-accumulating subpopulations, we hypothesized that the abnormal mitochondrial transfer between cardiomyocytes and surrounding resident cells may contribute to these phenotypes. TNTs, which mediate long-distance mitochondrial transfer, and Drp1, which is closely associated with mitochondrial quality control in cardiomyocytes during sepsis, attracted our attention. Next, we investigated the formation and extension of TNTs between cardiomyocytes and resident cells and their relationship with Drp1.

**Fig. 5.**
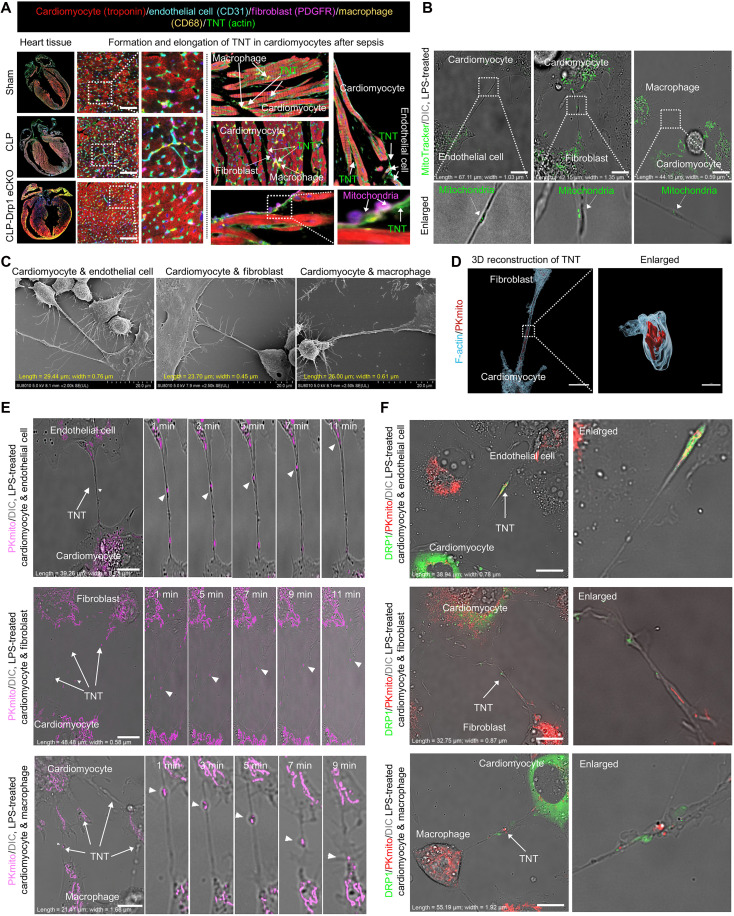
Super-high-resolution imaging of TNT formation and elongation and metastasis in damaged mitochondria between cardiomyocytes and cardiac resident cells after sepsis. (**A**) mIHC staining images showing TNT formation and extension and metastasis of damaged mitochondria in the heart tissues of mice in the Sham, CLP 24h, and CLP + Drp1 eCKO groups. Cardiomyocytes were labeled with troponin (red); endothelial cells were labeled with CD31 (cyan); fibroblasts were labeled with PDGFR (purple); macrophages were labeled with CD68 (yellow); and TNTs were labeled with actin (green); *n* = 6 per group. Scale bars, 25 μm. (**B**) Nanolive images showing the morphology of TNTs between the primary cardiomyocytes and the primary endothelial cells, fibroblasts, and macrophages after treated with LPS. Mitochondria were labeled with MitoTracker Green; *n* = 3 per group. Scale bars, 20 μm. (**C**) SEM images showing TNT formation of cardiomyocytes cocultured with endothelial cells, fibroblasts, and macrophages; *n* = 3 per group. Scale bars, 20 μm. (**D**) 3D reconstruction images showing the TNT structure suspended between cells; *n* = 3 per group. Scale bars, 15 μm. (**E**) Time-lapse recording of the metastasis of mitochondria via TNTs between primary cardiomyocytes and primary endothelial cells, fibroblasts, and macrophages by HIS-SIM after treated with LPS. Mitochondria were labeled with PKmito purple; *n* = 3 per group. Scale bars, 10 μm. (**F**) HIS-SIM images showing metastasis of mitochondria and Drp1 protein via TNTs between the primary cardiomyocytes and the primary endothelial cells, fibroblasts, and macrophages after treated with LPS. Mitochondria were labeled with PKmito; Drp1 protein was labeled with plasmids (green); *n* = 3 per group. Scale bars, 10 μm. eCKO, conditional knockout. DIC, differential interference contrast.

mIHC with antibodies against troponin, CD31, platelet-derived growth factor receptor (PDGFR), CD68, TOMM20, and F-actin was used to label cardiomyocytes, endothelial cells, fibroblasts, macrophages, mitochondria, and TNTs, respectively. We observed that, compared with control mice, septic hearts exhibited a significant increase in the number of TNTs between cardiomyocytes and surrounding resident cells, accompanied by a marked increase in both the average length and width of TNTs (Fig. 5A and fig. S6A). Consistent with mIHC results, Nanolive, scanning electron microscopy (SEM), three-dimensional (3D) structured illumination microscopy (HIS-SIM) imaging, and fluorescence recovery after photobleaching demonstrated that, after treated with lipopolysaccharide (LPS), cardiomyocytes extended abundant suspended TNT structures toward endothelial cells, fibroblasts, or macrophages (Fig. 5, B to D; fig. S6, B to D; and movies S1 to S4). Calcein-AM transfer assays combined with 3D HIS-SIM imaging further confirmed that these cardiomyocyte-driven TNTs have functional transport capacity, mediating intercellular transfer of “cargo” between cardiomyocytes and surrounding resident cells, including the translocation of mitochondria (Fig. 5E and fig. S6E).

Notably, in addition to the transfer of damaged mitochondria, intercellular transfer of Drp1 was also observed along TNTs connecting cardiomyocytes and resident cells (Fig. 5F, fig. S6F, and movies S5 to S7). These results suggest that cardiomyocytes transfer damaged mitochondria to resident cells via TNTs after treated with LPS, a process that potentially involves Drp1.

**Fig. 6.**
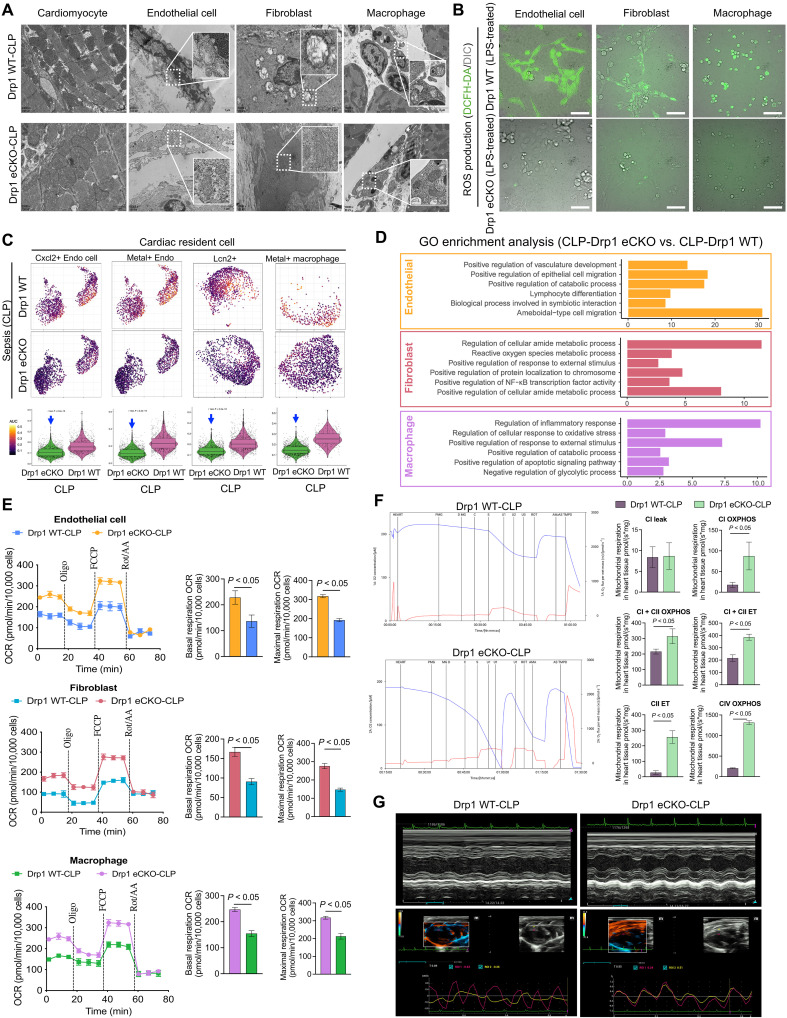
Drp1 mediates the functional transformation of cardiac resident cells after sepsis. (**A**) TEM images showing the morphology of cardiomyocytes, endothelial cells, fibroblasts, and macrophages in mouse heart tissue from the Drp1 WT-CLP and Drp1 eCKO-CLP mice; *n* = 8 per group. Scale bars, 2 μm. (**B**) ROS detection in primary endothelial cells, fibroblasts, and macrophages from the Drp1 WT-CLP and Drp1 eCKO-CLP mice after treated with LPS; *n* = 3 per group. Scale bars, 50 μm. (**C**) scRNA-seq analysis of Cxcl2+ endothelial cells, Metal+ endothelial cells, Lcn2+ fibroblasts, and Metal+ macrophages in Drp1 WT-CLP and Drp1 eCKO-CLP mice with cell distribution density, UMAP, and violin plots. (**D**) GO enrichment analysis of endothelial cells, fibroblasts, and macrophages after sepsis. (**E**) Mitochondrial respiration capacity assays and basal and maximal respiratory profiling of primary endothelial cells, fibroblasts, and macrophages from Drp1 WT-CLP and Drp1 eCKO-CLP mice; *n* = 8 per group. (**F**) Representative images of O_2_ concentration changes and O_2_ flux per mass and bar graphs from Drp1 WT-CLP and Drp1 eCKO-CLP mice. Oxygen consumption capacity measured by high-resolution respirometry in complexes I and II, including CI leak, CI OXPHOS, CI + CII OXPHOS, CI + CII ET, CII ET, and CIV OXPHOS; *n* = 8 per group. (**G**) Ultrasound and endocardial/epicardial strain coefficients from Drp1 WT-CLP and Drp1 eCKO-CLP mice; *n* = 8 per group. ET, electron transfer system.

To determine the role of Drp1 in TNT formation and extension, we generated cardiomyocyte-specific Drp1 conditional knockout mice (Drp1 Flox/Flox, Myh6 Cre/+) (fig. S7, A and B). These mice exhibited significantly less accumulation of damaged mitochondria in cardiomyocytes, endothelial cells, fibroblasts, and macrophages near cardiomyocytes than did Drp1 wild-type (WT) septic mice (Fig. 6A). Correspondingly, mIHC demonstrated a substantial reduction in TNT connections between cardiomyocytes and resident cells in Drp1 conditional knockout septic hearts (Fig. 5A).

DCFH-DA detection revealed that the ROS levels in cardiomyocytes, endothelial cells, fibroblasts, and macrophages were significantly lower in Drp1 knockout mice than in WT mice after treated with LPS (Fig. 6B and fig. S7C). Single-nucleus RNA sequencing of heart tissues showed that Drp1 deletion in cardiomyocytes attenuated functional transformation in resident cells. Notably, newly emerged subpopulations, including Cxcl2+ endothelial cells, Metal+ endothelial cells, Lcn2+ fibroblasts, and Metal+ macrophages, disappeared in Drp1 knockout mice (Fig. 6C). GO enrichment analysis revealed enhanced anti-inflammatory functions and improved mitochondrial metabolic capacity in the resident cells of Drp1 knockout mice (Fig. 6D). Seahorse metabolic assays confirmed significantly increased basal and maximal respiratory capacities in the cardiomyocytes, endothelial cells, fibroblasts, and macrophages of Drp1 knockout mice compared with those in WT mice (Fig. 6E and fig. S7D). In addition, high-resolution respirometry (Oroboros O2K system), ultrasound, and laser speckle imaging of cardiac tissues also reflected the protective effects of Drp1 knockout at the level of cardiac function (Fig. 6, F and G, and fig. S7E). Consistent with previous report, interference with Cdc42 expression in cardiomyocytes effectively suppresses TNT (*17*). However, inhibition of TNT formation between cardiomyocytes and surrounding resident cells still fails to significantly improve cardiac function in septic mice. (fig. S8).

These findings indicate that cardiomyocytes promote TNT formation and extension during sepsis, transferring damaged mitochondria to the surrounding resident cells, thereby inducing the emergence of dysfunctional subpopulations. Specific Drp1 deletion in cardiomyocytes disrupted TNT formation, highlighting the role of Drp1 in this process.

### Drp1 regulates cytoskeletal remodeling to facilitate TNT formation and mitochondrial transfer

**Fig. 7.**
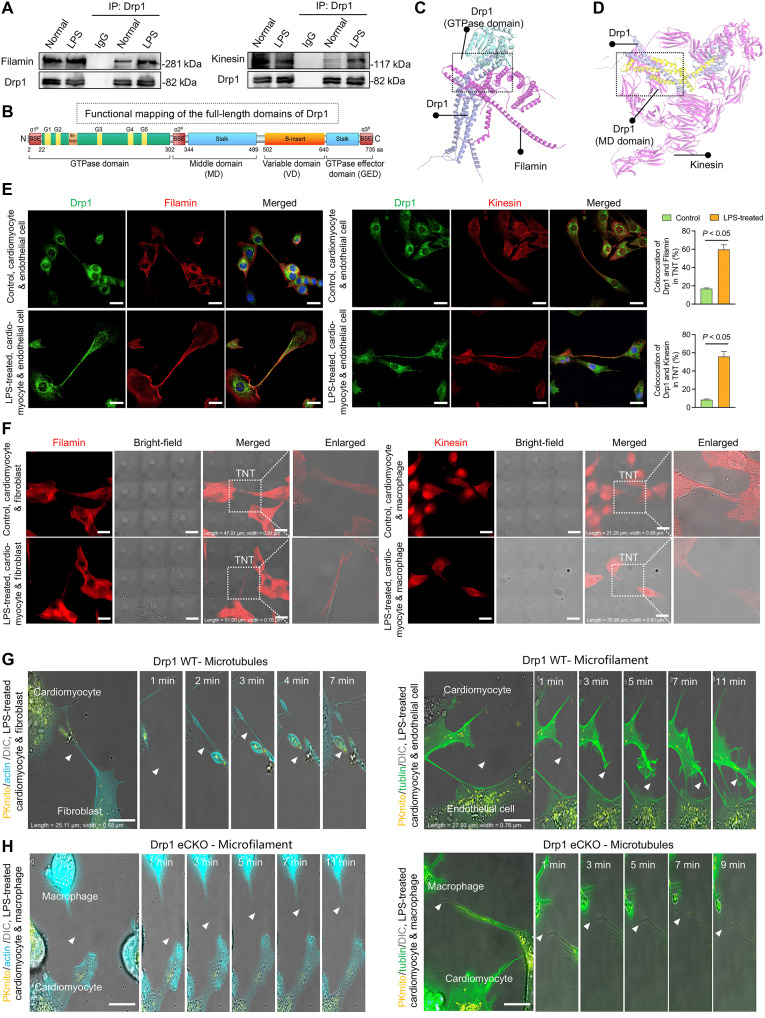
Drp1 influences the formation and elongation of TNTs through interactions with Filamin and Kinesin proteins. (**A**) Western blot analysis and Co-IP assay showing the Drp1 and Filamin/Kinesin binding ability of the myocardial tissue after CLP; *n* = 8 per group. (**B**) Functional pattern of the full-length domain of Drp1 protein. (**C** and **D**) Schematic diagram of the docking of Filamin and Kinesin with Drp1. (**E**) Immunofluorescence images showing the expression and distribution of Drp1 (green) and Filamin/Kinesin (red) in cardiomyocytes cocultured with endothelial cells from control and LPS-treated groups; *n* = 3 per group. Scale bars, 25 μm. (**F**) Immunofluorescence images showing the expression of Filamin/Kinesin (red) in cardiomyocytes cocultured with fibroblasts or macrophages; *n* = 3 per group. Scale bars, 25 μm. (**G** and **H**) Time-lapse recordings of mitochondrial transfer via TNT between WT and Drp1 eCKO primary cardiomyocytes and primary endothelial cells, fibroblasts, and macrophages by HIS-SIM. Mitochondria were labeled with PKmito (yellow); TNTs were labeled with tubulin (green) and actin (cyan); *n* = 3 per group. Scale bars, 10 μm. IgG, immunoglobulin G; GTPase, guanosine triphosphatase; aa, amino acids.

The formation and extension of TNTs heavily rely on the dynamic remodeling of actin and microtubule cytoskeletons. Filamin, an actin–cross-linking protein, connects actin filaments to construct an internal actin network within TNTs. During TNT extension, cell membrane protrusions are stabilized by microtubules and further elongated by motor proteins such as Kinesin, forming tubular structures that connect cells (*18*). Previous studies have reported that Drp1 regulates the expression of cytoskeletal regulatory proteins by altering their activation states (*19*). Coimmunoprecipitation (Co-IP) experiments showed that LPS treatment significantly enhanced the interaction between Drp1, Filamin, and Kinesin in cardiomyocytes (Fig. 7A). Purified Drp1 recombinant protein chip screening confirmed direct interactions between Drp1 and both Filamin and Kinesin (fig. S9A), suggesting that Drp1 influences TNT formation and extension via these interactions. To identify the binding domains, we used the Protein Data Bank to retrieve the 3D structures of Drp1, Filamin, and Kinesin, supplemented the missing regions with AlphaFold data, and performed structural modeling and global molecular docking analysis using H-Dock. The results indicated that Drp1 interacts with Filamin with a binding free energy of −16.2 kcal/mol, with the potential binding site located on the guanosine triphosphatase domain of Drp1 (Fig. 7, B and C, and fig. S9B). Similarly, Drp1 interacts with Kinesin with a binding free energy of −12.8 kcal/mol, with the potential binding site on the MD domain of Drp1 (Fig. 7D and fig. S9C). In addition, fluorescence labeling of Drp1 together with Filamin or Kinesin in cells within the coculture system revealed that LPS treatment markedly enhanced the colocalization of Drp1 with Filamin or Kinesin inside intercellular TNTs (Fig. 7E). SIM imaging further demonstrated that, following LPS stimulation, the distribution pattern of Kinesin or Filamin within TNTs shifted from an originally uniform arrangement along the TNT fibers to a discontinuous, misaligned, or even clustered aggregation, reflecting disruptions in both cytoskeletal support and cargo transport pathways (Fig. 7F). Collectively, these results supported our hypothesis that under septic stress, Drp1 interacts with Kinesin and Filamin to drive abnormal cytoskeletal remodeling of TNTs, thereby impairing their stability and function.

To further investigate the role of Drp1 in cytoskeletal remodeling, we isolated cardiomyocytes from Drp1 conditional knockout and WT mice and cultured them in vitro. Actin and tubulin plasmids were used to label microfilaments and microtubules, respectively, and SIM was used for the long-term observation of TNT formation and extension between cardiomyocytes and resident cells. Upon LPS treatment, microfilaments and microtubules in WT cardiomyocytes exhibited dynamic outward extension over time, forming “pseudopodia” that gradually established TNTs. These TNTs mediated the transfer of intact mitochondria to the surrounding resident cells (Fig. 7G and movies S8 and S9). In contrast, cardiomyocytes from Drp1 conditional knockout mice failed to form functional TNTs capable of transferring mitochondria, as their pseudopodia extensions remained incomplete (Fig. 7H and movies S10 and S11). These findings suggest that Drp1 promotes TNT formation by regulating actin and microtubule interactions and plays a crucial role in the dynamic extension of these cytoskeletal elements. By modulating these remodeling processes, Drp1 ensures the structural integrity and functionality of TNTs, enabling the efficient transfer of mitochondria and other organelles to support intercellular communication and material exchange.

## DISCUSSION

**Fig. 8.**
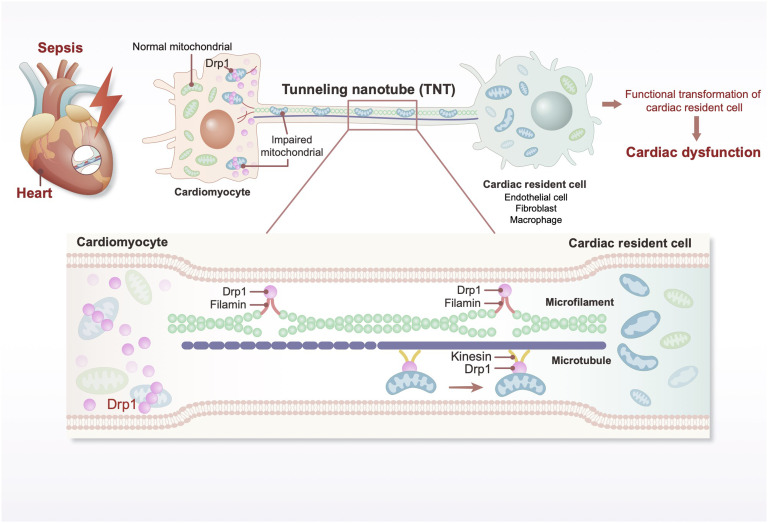
Mechanistic illustration of Drp1-mediated TNT formation and mitochondrial transfer in postsepsis cardiac dysfunction. In sepsis, the conformational alteration in Drp1 in cardiomyocytes interacts with Filamin and Kinesin proteins, facilitating the formation and elongation of TNTs between cardiomyocytes and the surrounding resident cells. This process enables the transfer of damaged mitochondria from cardiomyocytes to the adjacent resident cells via TNTs, thereby contributing to postsepsis cardiac dysfunction.

Sepsis-induced cardiac dysfunction considerably affects patient outcomes and involves multifactorial and multiprocess interactions. Existing studies have predominantly focused on cardiomyocyte damage and repair; however, they do not fully elucidate the intricate pathological processes underlying septic cardiac dysfunction. To address this gap, we used scRNA-seq to comprehensively analyze the distribution and functional phenotypes of cardiac cell subpopulations after sepsis. In addition, we investigated the role of Drp1-mediated TNT cytoskeletal remodeling in facilitating the transfer of damaged mitochondria from cardiomyocytes to the surrounding resident cells (Fig. 8).

Previous studies on intercellular mitochondrial communication have primarily emphasized indirect mechanisms such as the release and uptake of mitochondrial microvesicles (*20*). Owing to technological limitations, the study of direct communication mechanisms, such as TNT-mediated mitochondrial transfer, has progressed relatively slowly (*11*). However, the dense structure and tightly packed cells in the cardiac tissue highlight the importance of direct intercellular communication. Using SIM super-resolution imaging, we directly and longitudinally observed TNT-mediated mitochondrial transfer between cardiomyocytes and resident cells during sepsis-associated cardiac dysfunction. This process plays a critical role in the progression of cardiac dysfunction and is closely associated with cytoskeletal dynamics.

The cytoskeleton, a vital subcellular structure in eukaryotic cells, maintains cell morphology and mediates intracellular and intercellular material and signal transport (*21*, *22*). Beyond providing structural support, cytoskeletal remodeling dynamically regulates organelle positioning, interactions, and functions. Under normal conditions, limited ER-mitochondrion interactions maintain cellular energy supplies, mitochondrial autophagy, and calcium homeostasis. However, abnormal cytoskeletal distribution can lead to excessive ER-mitochondrion interactions, resulting in mitochondrial hyperfragmentation and damage (*16*). The cytoskeleton also mediates intercellular communication through TNTs, facilitating the exchange of mitochondria, metabolic products, and genetic information (*23*).

For example, tumor cells exploit TNTs to “steal” functional mitochondria from immune cells, thereby enhancing their metabolic capacity and invasiveness (*24*). Similarly, TNTs between pericytes in retinal capillaries coordinate neurovascular coupling, with TNT ablation disrupting calcium flow and vascular responses (*25*). These examples underscore the importance of cytoskeletal remodeling in maintaining TNT functionality and facilitating material exchange. In this study, we observed that TNT-mediated mitochondrial communication between cardiomyocytes and resident cells is closely linked to cytoskeletal remodeling and is regulated by Drp1 conformational changes.

As terminally differentiated cells, cardiomyocytes must efficiently eliminate damaged mitochondria to maintain functional integrity. Previous studies have demonstrated that restoring and preserving mitochondrial homeostasis in cardiomyocytes not only alleviates cellular dysfunction but also significantly attenuates cardiac impairment associated with sepsis (*4*, *26*, *27*). Our findings indicated that after sepsis, cardiomyocytes actively generate TNTs to communicate with cardiac resident cells and “throw” damaged mitochondria through these structures. This mitochondrial transfer contributed to the mitigation of cardiomyocytes. The activation of this mechanism represents an intrinsic protective response, the primary objective of which is to rescue injured cardiomyocytes and prevent secondary damage. However, persistent export of dysfunctional mitochondria from cardiomyocytes to cardiac resident cells may exacerbate injury in these cells. Notably, as bidirectional intercellular conduits, TNTs not only facilitate the release of cellular components but may also enable the uptake of exogenous materials (*28*). However, current methodologies remain limited in their capacity to accurately assess the quality and directional transport of mitochondria via TNTs, which poses a significant challenge in fully elucidating the role of TNT-mediated mitochondrial transfer in the recovery from septic myocardial injury (*29*–*31*).

Drp1 undergoes phosphorylation and dephosphorylation under ischemia/hypoxia, promoting mitochondrial hyperfragmentation and oxidative stress, leading to mitochondrial dysfunction and cell death (*15*, *32*). Accordingly, targeting Drp1 or related pathways has been proposed as a strategy for mitigating mitochondrial dysfunction (*33*) and cellular damage (*34*, *35*). In addition to mitochondrial regulation, the interactions of Drp1 with the cytoskeleton have become a popular research topic. For instance, Inverted formin 2 (INF2)–mediated actin polymerization in the ER recruits Drp1, enhances ER-mitochondrion interactions, and drives mitochondrial fission under pathological conditions (*16*). In acute kidney injury, Drp1 activation induces mitochondrial fragmentation and actin disruption, thereby impairing organelle communication and distribution (*36*). In this study, we demonstrated that Drp1 conformational changes facilitate TNT formation by interacting with Filamin and Kinesin. Drp1 regulates the dynamic extension of microfilaments and microtubules, ensuring TNT structural integrity and functionality for mitochondrial transfer between cells. These findings highlight a novel mechanism of Drp1-mediated mitochondrial communication during sepsis.

Despite these insights, our study has several limitations. First, the CLP mouse model used to simulate sepsis may not fully replicate the human sepsis pathophysiology, and further clinical validation is needed. Second, the small sample size (two mice per group for scRNA-seq) may have limited the detection of all potential subpopulations and functional changes. Future studies should increase the sample size to improve statistical reliability and generalizability. Last, although scRNA-seq provides comprehensive gene expression profiles, it lacks spatial resolution. As the cellular location within the heart influences intercellular communication, integrating spatial transcriptomics in future studies would enable the correlation of cellular interactions with spatial context, offering a more complete understanding of sepsis-induced cardiac damage.

This study revealed that Drp1 activity and conformational changes play a central regulatory role in the intercellular transfer of damaged mitochondria following sepsis, driving the functional transformation of various cardiac resident cells and contributing to the progression of septic cardiac dysfunction. These findings offer new insights and potential therapeutic targets for improving septic cardiac function by modulating the intercellular communication in clinical practice.

## MATERIALS AND METHODS

### Animal model establishment and treatment

C57BL/6J male WT mice (7 to 8 weeks old, weighing 20 to 25 g) were obtained from the Laboratory Animal Center of Chongqing Medical University. Myocardial-specific Drp1-knockout mice (Drp1 eCKO, Drp1 Flox/Flox; Myh6 Cre+) were generated using CRISPR-Cas9 technology (Shanghai Model Organisms Center Inc., Shanghai, China). All mice were maintained in a specific pathogen–free environment under a 12-hour light-dark cycle with unrestricted access to food and water. All animal experiments were approved by the Institutional Animal Care and Use Committee of the Second Affiliated Hospital of Chongqing Medical University (approval no. IACUC-SAHCQMU-2025-0122) and conducted in accordance with institutional guidelines and the National Institutes of Health Guide for the Care and Use of Laboratory Animals.

A mouse model of sepsis was established using CLP as described previously (*7*). Mice were anesthetized via intraperitoneal injection of pentobarbital sodium (45 mg/kg) and positioned supine on the surgical platform. Abdominal hair was removed, and a 1.5-cm midline incision was made to expose the cecum. The cecum was ligated ~0.7 cm from its distal end using a 21 G triangular needle, and the fecal matter was allowed to leak into the abdominal cavity. Both omental tissues were carefully removed, and the incision was sutured in layers. Postoperative care included subcutaneous administration of buprenorphine (0.05 mg/kg) for analgesia and sterile saline for fluid replacement. The mice were closely monitored to ensure recovery and manage potential complications associated with the procedure.

Adeno-associated virus (AAV) vectors for Cdc42 knockdown were constructed and produced by OBiO Technology Corp. Ltd. (Shanghai, China). The MyoAAV1A serotype was used, and the cardiac troponin T (cTnT) promoter was used to achieve cardiomyocyte-specific expression. The specific vectors used in this study were pcAAV-cTnT-miR30shRNA (NC)-WPRE and pcAAV-cTnT-miR30shRNA (Cdc42)-WPRE, with the targeting sequence GCTGTTGGTAAAACCTCTTAA. Fourteen days before the induction of the CLP model, mice received tail vein injections of 50 μl of AAV carrying Cdc42 short hairpin RNA (shRNA), with a viral titer of 2.61 × 10^13^ vg/ml. All animal experiments were approved by the appropriate ethics committee.

### Cell model establishment and processing

Primary vascular endothelial cells (VECs), CFs, macrophages, and cardiomyocytes were isolated following established protocols (*12*, *37*, *38*). VECs were isolated from the thoracic aortae of C57BL/6J mice. After anesthesia and ethanol sterilization, the thoracic cavity was opened to expose the heart and aorta, and the surface adipose tissue was carefully removed. The aorta was cut into 1 mm–by–1 mm fragments and digested with a solution containing 0.25% trypsin (Gibco, 25300120) and 2% type II collagenase (Sigma-Aldrich, C2-28). The digested tissue was filtered through a 400-mesh sieve to remove undigested fragments. The resulting cell suspension was centrifuged and seeded in endothelial growth medium containing 20% fetal bovine serum (FBS; Gibco, A5670701), endothelial cell growth supplement (0.05 mg/ml), and 1% penicillin-streptomycin. After incubation at 37°C in a 5% CO_2_ atmosphere for 4 hours, nonadherent and loosely adherent cells were removed, and adherent cells were cultured to obtain primary VECs. CFs and cardiomyocytes were isolated from the hearts of neonatal C57BL/6J mice. The hearts were collected after anesthesia, washed in sterile phosphate-buffered saline (PBS; Gibco, 10010023), and cut into 1 mm–by–1 mm pieces. Digestion was performed sequentially with 0.25% trypsin and 2% type II collagenase, and the process was terminated using Dulbecco’s modified Eagle’s medium (DMEM; Gibco, 11965092) supplemented with 25% horse serum (Gibco, 16050122). The cell suspension was filtered through a 400-mesh sieve to remove the undigested tissue. The filtrate was resuspended in DMEM containing 5% FBS, 10% horse serum, and 1% penicillin-streptomycin. CFs were allowed to adhere for 2 hours at 37°C in a 5% CO_2_ incubator, after which nonadherent cells were collected for cardiomyocyte isolation. CFs were cultured in DMEM containing 10% FBS and 1% penicillin-streptomycin, whereas cardiomyocytes were seeded onto 0.1% gelatin-coated dishes and cultured in DMEM containing 5% FBS, 10% horse serum, and 1% penicillin-streptomycin. Cardiac macrophages were isolated from adult C57BL/6J mice. Hearts were collected after anesthesia, washed in sterile PBS, and digested in a solution containing 0.25% trypsin, 2% type II collagenase, and deoxyribonuclease I (0.1 mg/ml; Sigma-Aldrich, AMPD1) at 37°C for 30 min. The digested tissue was filtered through a 200-mesh sieve, and red blood cells were lysed using erythrocyte lysis buffer. The resulting single-cell suspension was incubated with fluorescein isothiocyanate–labeled CD68 antibody (1:1000; BioLegend, 137005) at 4°C for 30 min. Macrophages were sorted by flow cytometry and cultured in RPMI 1640 medium (Gibco, 61870036) containing 10% FBS and macrophage colony-stimulating factor (10 ng/ml). To simulate sepsis, cells were treated with LPS (10 μg/ml) in DMEM for 24 hours, while untreated cells were cultured in DMEM as controls. To visualize intercellular interactions, multiple adenoviral constructs were generated to label different cell types (OBiO Technology, Shanghai). Specifically, cardiomyocytes were transduced with Ad-cTnT-GFP; meanwhile, endothelial cells, fibroblasts, and macrophages were transduced with cell type–specific adenoviruses Ad-mFLT1-mCherry, Ad-Col1a-mCherry, and Ad-CD68-mCherry, respectively. In some experiments, cardiomyocytes were alternatively labeled with 10 μM Calcein-AM. Labeled cells were subsequently used for coculture experiments to track cellular communication and organelle transfer (movies S12 to S14).

### Isolation of primary neonatal mouse cardiomyocytes

Isolation of primary cardiomyocytes was performed as described previously (*39*). Primary cardiomyocytes were isolated from 1- to 3-day-old C57BL/6 neonatal mice. Briefly, neonatal mice were disinfected with 75% ethanol, and hearts were rapidly excised under sterile conditions into ice-cold D-Hanks or PBS. Residual blood was removed by gentle squeezing, and hearts were minced into small fragments. Tissue pieces were incubated overnight at 4°C in 0.25% trypsin. The next morning, trypsin was removed and digestion was terminated with an equal volume of DMEM containing 10% FBS. The tissue was then subjected to repeated digestion cycles in 0.08% type II collagenase at 37°C (8 to 15 min each) until fully dissociated. Neutralized cell suspensions from each cycle were pooled, filtered through a 40-μm strainer, and centrifuged at 800 rpm for 5 min. The pellet was resuspended in DMEM with 10% FBS and preplated for 1.5 to 2 hours to remove noncardiomyocytes. The unattached cardiomyocytes were collected, counted, and seeded at appropriate densities for culture. Medium was changed after 48 hours to allow stable attachment.

### scRNA-seq and data processing

Single-cell suspensions were processed using the Chromium Single Cell 3′ Reagent Kit v.3 (10x Genomics, Pleasanton, CA, USA) (*40*) for capture, RNA barcoding, cDNA amplification, and library construction, followed by sequencing on the NovaSeq platform (Novel Bioinformatics Ltd. Co. Shanghai, China). The resulting expression data were aligned to the GRCm38 reference genome using CellRanger v.4.0. and then processed in R (v4.2) with the “Seurat” (v5.1.0) package (*41*) for quality control, normalization, and integration. Cell types were filtered on the basis of gene count, unique molecular identifier (UMI) count, and mitochondrial content, followed by the removal of doublets using “ScrubletR” (*42*).

### Cell clustering, annotation, and subset analysis

Cell clustering and subset analysis were performed using the Seurat (v.5.1.0) package (*41*). Initially, the “FindNeighbor” function was used to compute the similarity of the first 30 principal components, followed by clustering into 21 populations with a resolution of 0.5 using the “FindCluster” function. Marker genes were identified using the bimodal likelihood ratio test via the “FindAllMarkers” function, selecting genes with expression in more than 25% of cells and a mean log fold change greater than 0.5. The top 10 significant genes were annotated on the basis of classical markers and the PanglaoDB database (*43*). The cells were classified into 10 categories: endothelial cells, fibroblasts, smooth muscle cells, cardiomyocytes, monocytes, macrophages, neutrophils, T cells, B cells, and Schwann cells. Marker gene visualization was performed using “ComplexHeatmap” (v.2.22.0) (*44*), while the “ggplot2” (v.3.5.1) package was used to depict cell class proportions and marker genes. UMAP was performed using the “DimPlot” function.

For subset analysis, key populations, including endothelial cells, fibroblasts, and monocytes/macrophages, were isolated using the “subset” function. Variable genes (2000) within each subset were identified using “FindVariableFeatures,” and batch effects were corrected with “Harmony” (v.1.2.3) (*45*). Cells were reclustered at a resolution of 0.2, and UMAP plots of the first 10 principal components were generated. Differentially expressed genes (DEGs) in each subset were identified using the biomodal method with the FindAllMarkers function, with thresholds of log fold change > 0.25 and *P* < 0.05. The subsets were annotated on the basis of the top 10 DEGs with the most significant differences. Visualization was conducted in 2D using the DimPlot function and in 3D with the “scatterplot3d” (v.0.3.44) package. The “Nebulosa” (v.1.16.0) package (*46*) was used for marker gene and cell proportion visualization.

### Functional enrichment and pseudotime trajectory analysis

Feature enrichment analysis was conducted to identify the biological functions of DEGs using the “ClusterProfiler” (v.4.14.4) package (*47*). The “enrichGO” function within the “compareCluster” function was used to analyze biological processes, with gene IDs mapped via the “org.Mm.eg.db” (v.3.20.0) package. Mouse HALLMARK gene sets were obtained using the “msigdbr” (v.7.5.1) package (*48*), and functional differences across subsets were evaluated using the “GSVA” (v.2.0.4) package (*49*) with the Gaussian method. Results were visualized using the ComplexHeatmap (v.2.22.0) package.

Pseudotime trajectory analysis was performed to investigate the cellular state transitions using the “monocle3” (v.2.34.0) and “CytoTRACE” (v.0.3.3) packages (*50*). Subpopulation cells were isolated using the subset function, and a standard cell dataset object was created using the “newCellDataSet,” “estimateSizeFactors,” and “estimateDispersions” functions for normalization and dispersion evaluation. Genes with an average expression greater than 0.1 were identified using the “dispersionTable” function and designated as ordering genes using the “setOrderingFilter” function. Dimensionality reduction was performed using the DDRtree method in the “reduceDimension” function, and the “orderCell” function was applied to construct pseudotime trajectories. Trajectories were visualized with the “plot_cell_trajectory” function.

### TF activity analysis

TF analysis was performed using the “SCENIC” (v.1.3.1) package (*51*). The mouse TF database (mm10) was initialized with the “initializeScenic” function, and correlations between TFs and single-cell transcriptome genes were computed using the “runCorrelation” function. The “GENIE3” algorithm inferred regulatory networks, while the “RcisTarget” algorithm identified enriched TFs. Regulatory network activity was quantified using the “AUC cell” algorithm. Results were visualized using the “calcRSS” function and the ComplexHeatmap (v.2.22.0) package.

### Cell flow cytometry and fluorescence-activated cell sorting

For subtypes obtained from scRNA-seq, we used cell flow cytometry to sort the cells for postculture. Briefly, VECs and CFs were digested with 0.25% trypsin, and bone marrow–derived macrophages (BMDMs) were prepared in a single-cell suspension with a cell scraper. Primary antibodies against Aqp7 (1:50; IPODIX, IPDX12462), Cxcl2 (1:50; Proteintech, 98259-1-RR), and Metal (1:50; Abcam, ab12228); Lcn2 (1:50; Proteintech, 26991-1-AP) and Fmo2 (1:50; IPODIX, IPDX10542); and Hpgd (1:50; Abcam, ab187160), Ccr2 (1:50; BioLegend, 150607), and Metal were incubated with the VEC, CF, and BMDM single-cell suspensions, respectively, for 30 min at 4°C and then washed with PBS. The target cells were automatically sorted according to the fluorescence intensity of the markers using a BD FACSDiscover S8 Spectral Sorter (BD Biosciences). Target cells were collected for further functional assays.

### Multiplex fluorescence immunohistochemical staining

Paraffin-embedded heart sections (5 μm) were deparaffinized, rehydrated through an ethanol gradient, and subjected to antigen retrieval using sodium citrate buffer (Servicebio, China). After blocking with goat serum, sections were incubated overnight at 4°C with primary antibodies [Cldn5 (1:200; Cell Signaling, #66879), Metal (:200; Abcam, ab12228), Cxcl2 (1:200; Abcam, ab317569), Gsn (1:200; Proteintech, 11644-2-AP), Fmo2 (1:200; Proteintech, 67019-1-Ig), Lcn2 (1:200; Proteintech, 26991-1-AP), CD74 (1:200; Abcam, ab289885), troponin (1:200; Abcam, ab209813), CD31 (1:200; Abcam, ab182981), PDGFR (1:200; Abcam, ab203491), CD68 (1:200; Abcam, ab283654), and actin (1:200; Abcam, ab8227)]. The following day, horseradish peroxidase polymer–labeled secondary antibodies (1:200; Abcam, ab6721) were applied, followed by tyramide signal amplification (TSA) with TG-series tyramide fluorophores (TG 520N, TG 570N, TG 620N, TG 760N, and TG 700N) for signal amplification. Microwave treatment with sodium citrate buffer was performed after each staining cycle to remove residual antibody complexes. Sections were counterstained with 4′,6-diamidino-2-phenylindole (DAPI; Thermo Fisher Scientific, P36971), mounted in antifluorescence quenching medium (Thermo Fisher Scientific, USA), and scanned using a TissueFAXS quantitative analyzer (TissueGnostics, Austria).

### RNA isolation and reverse transcription polymerase chain reaction

Total RNA was extracted from the mouse heart samples using a Total RNA Extraction Kit (Thermo Fisher Scientific, AM1931) following the manufacturer’s protocol. RNA (1 μg) was reverse transcribed using a Fast RT Reagent Kit (Clontech, Japan). Quantitative real-time polymerase chain reaction was performed using the FastStart Essential DNA Green Master (Roche, 06924204001), with β-actin as the internal control. Data were analyzed using CFX Maestro v.2.3 (Bio-Rad, USA), with each sample run in triplicate.

### Coimmunoprecipitation

Mouse heart lysates were prepared in radioimmunoprecipitation assay buffer (MeilunBio, MA0151) containing protease and phosphatase inhibitors (Beyotime). Equal lysate amounts were incubated overnight with Drp1 antibody (1:50; Novus Biologicals, NB110-55288) or immunoglobulin G (1:50; Cell Signaling, #2729) at 4°C, followed by incubation with protein A/G magnetic beads (MedChemExpress, HY-K0202) for 6 hours. After washing, the immune complexes were eluted with SDS buffer, boiled, and subjected to SDS–polyacrylamide gel electrophoresis and with polyvinylidene difluoride transfer. Membranes were probed with Drp1, Filamin, and Kinesin antibodies and analyzed using Quantity One V.4.62 and the ImageJ software. Protein expression was normalized to the control protein density.

### Cardiac ultrasound imaging and strain analysis

Cardiac function was assessed in septic mice using a high-resolution ultrasound system (Mindray Animalcare Co., Shen Zhen, China). Mice were anesthetized with 1.5% isoflurane in oxygen and placed on an ultrasound platform. 2D B- and M-mode echocardiographic images were obtained along the parasternal long and short axes. Left ventricular end-diastolic internal diameter and end-systolic internal diameter were measured. Speckle-tracking echocardiography was applied to track both endocardial and epicardial motions, and the strain of the left ventricular anterior wall was calculated to assess ventricular motion and function. Ultrasound gel was used to ensure optimal acoustic coupling. After imaging, mice were monitored on a 37°C warming pad until full recovery from anesthesia.

### Laser speckle contrast imaging of the beating mouse heart

In this study, myocardial perfusion in mice was monitored in real time using a laser speckle contrast imaging system (RFLSI-ZW, RWD Life Science, China). The imaging head was positioned at a fixed distance of ~20 to 25 cm above the heart, and the focal distance was adjusted to obtain a clear image. The charge-coupled device camera exposure time was set to 20 ms. For each measurement, 30 consecutive images were acquired at 1-s intervals. Raw images were processed using the manufacturer’s software, and regions of interest were selected to calculate the blood flow index.

### Hematoxylin and eosin staining

The heart tissues were fixed in 4% paraformaldehyde for 24 hours, dehydrated using an ethanol gradient, embedded in paraffin, and sectioned (6 μm). Sections were deparaffinized, hydrated, and stained using an hematoxylin and eosin (H&E) staining kit (Solarbio, G1120). Images were captured at ×200 magnification using a CYTATION5 imaging system (Biotek-Agilent, USA).

### Transmission electron microscopy

Heart tissues were fixed in 2.5% glutaraldehyde at 4°C, washed in phosphate buffer, and postfixed in 1% osmium tetroxide. Dehydration was performed using an ethanol gradient and tert-butanol followed by CO_2_ drying. The samples were sectioned (50 nm), stained with uranium acetate and lead citrate, sputter-coated, and imaged using an H-7500 transmission electron microscope (Hitachi, Japan).

### ROS detection in cell lines and tissue

The level of intracellular and tissue ROS was evaluated using DCFH-DA staining (Beyotime, S30033S). For cell-based assays, cells at 70 to 80% confluence were incubated with 10 μM DCFH-DA at 37°C for 30 min, followed by PBS washing. For tissue analysis, freshly excised myocardial samples were rapidly frozen in liquid nitrogen, cryosectioned into 8- to 10-μm slices, and stained with 10 μM DCFH-DA at 37°C for 30 min in the dark. After two washes, nuclei were counterstained with a Hoechst 33342 kit, and the sections were sealed with an antifade mounting medium. Both cell and tissue samples were subsequently imaged using an Olympus SPIN SR confocal microscope (Japan) with 488-nm excitation and 501- to 563-nm emission, and fluorescence intensity was quantified using ImageJ software.

### Mitochondrial respiratory function testing

Mitochondrial respiration was assessed using an XF Cell Mito Stress Test Kit (Agilent, 103016, USA). Cells were seeded in XF plates at 70 to 80% confluence, followed by the sequential addition of 20 mM glutamate, 4 mM malate, and 1.5 mM adenosine diphosphate (ADP) to measure complex I respiration. Succinate (10 mM) was added to assess the complex II respiration. Carbonyl cyanide *p*-trifluoromethoxyphenylhydrazone (FCCP; 2.5 μM) was used to determine maximal uncoupled respiratory capacity, and rotenone (1 μM) evaluated complex II electron transfer system (ETS) activity. Antimycin A was added to measure the residual oxygen consumption. Data acquisition was performed on the Seahorse XF HS Mini Analyzer, and results were analyzed using Wave Desktop software (Agilent Technologies) and GraphPad Prism 9.

Cardiac mitochondrial respiratory function was assessed using an Oxygraph-2K (Oroboros, Innsbruck, Austria) system. Briefly, heart tissues (10 mg) were homogenized on ice in 400 μl of mitochondrial respiration medium, and 40 μl of the homogenate was applied for analysis. The substrate-inhibitor titration protocol was performed as follows: Complex I–dependent LEAK (proton leak) respiration was induced with 5 mM pyruvate, 10 mM glutamate, and 2 mM malate, followed by addition of 1 mM ADP to determine complex I oxidative phosphorylation (OXPHOS) capacity. Outer membrane integrity was tested by cytochrome c (10 μM). Subsequent addition of succinate (10 mM) enabled assessment of combined complex I + II OXPHOS capacity. ETS capacity was measured with FCCP (0.5 μM), and complex II–specific ETS activity was determined after rotenone (0.5 μM) injection. Antimycin A (2.5 μM) was then applied to inhibit complex III, and complex IV activity was quantified with 2 mM ascorbate plus 0.5 mM TMPD (*N*,*N*,*N*′,*N*′-tetramethyl-*p*-phenylenediamine). Oxygen flux was normalized to tissue weight.

### Measurement of reduced glutathione and oxidized glutathione

The levels of glutathione (GSH) and oxidized glutathione (GSSG) in heart tissue were determined using the GSH Content Assay Kit (Solarbio, BC1175) and the GSSG Content Assay Kit (Solarbio, BC1185), respectively. Briefly, ~0.1 g of fresh heart tissue was homogenized in 1 ml of extraction buffer. The homogenate was centrifuged at 800*g* for 10 min at 4°C, and the resulting supernatant was used for GSH or GSSG measurement according to the manufacturer’s instructions. Results were normalized to tissue weight and expressed as micrograms of GSH or GSSG per gram of fresh heart tissue.

### Live-cell imaging

Primary cardiomyocytes, endothelial cells, fibroblasts, and macrophages were cocultured in confocal dishes for 24 hours. Drp1, actin, and tubulin were labeled by plasmid transfection, and mitochondria were labeled using MitoTracker or PK Mito (Genvivo, PKMDR). Live-cell imaging was performed using a 3D Cell Explorer microscope (Nanolive) with structured illumination microscopy–optical diffraction tomography. Cells were maintained at 37°C in a 5% CO_2_ atmosphere. Image reconstruction and sparse deconvolution were performed as described previously (*52*).

### Small-molecule microarray screening

Small-molecule microarrays were developed to identify compounds that interact with Drp1 based on a previous method (*53*). A library of 3000 small molecules from a Food and Drug Administration–approved database was prepared on isocyanate-coated slides. Biotinylated Drp1 recombinant protein was incubated with the slides, followed by incubation with Cyanine 5 (Cy5)–labeled streptavidin. Fluorescence was detected using a LuxScanTM 10 K-A microarray scanner (Boao Biotech, Beijing, China), and data were analyzed using GenePix Pro v6.0. Positive interactions were identified by a fold change ≥ 1.5 and *P* < 0.05.

### Molecular docking

Molecular docking analysis of Drp1 with small molecules was performed using H-dock software. The structures of compounds such as Filamin and Kinesin were retrieved from the PubChem database. Homologous sequences of the receptor and ligand were identified through sequence similarity searches in the Protein Data Bank, followed by 3D structural modeling using MODELLER and sequence alignment using Clustal. Global molecular docking was performed, and the optimal model was selected on the basis of docking scores and active site data. The docking results were visualized using Discovery Studio software.

### Statistical analysis

All statistical analyses and graphing were performed using GraphPad Prism 10.2.3 (GraphPad Software Inc., USA) and SPSS (version 20.0; SPSS Inc., Chicago, IL, USA). Data showing a normal distribution and homogeneity of variance were expressed as mean ± SD, and multiple comparisons were performed using a two-sided unpaired Student’s *t* test or one-way analysis of variance (ANOVA), followed by Tukey’s post hoc test. For cases of nonnormal distribution and irregular variance, multiple comparisons were performed using the Mann-Whitney *U* test or Kruskal-Wallis test, followed by Tukey’s post hoc test. Statistical significance was defined as a two-tailed *P* value of less than 0.05.
