# Supplementary material for: Cytoskeletal remodeling promotes tunneling nanotube formation and drives cardiac resident cell mitochondrial transfer in sepsis
Source: Sci Adv. 2026 Mar 11;12(11):eadz3266. doi: 10.1126/sciadv.adz3266 (PMC12978241; doi:10.1126/sciadv.adz3266)
Supplement: Supplementary file 1 — Figs. S1 to S9 Legends for movies S1 to S14 Legend for table S1 [file sciadv.adz3266_sm.pdf]

Science Advances
AAAS

Supplementary Materials for

Cytoskeletal remodeling promotes tunneling nanotube formation and drives cardiac resident cell mitochondrial transfer in sepsis

Rui Song *et al.*

Corresponding author: Chenyang Duan, duanchenyang1991@cqmu.edu.cn; João Conde, joao.conde@nms.unl

Sci. Adv. **12**, eadz3266 (2026)
DOI: 10.1126/sciadv.adz3266

The PDF file includes:

Figs. S1 to S9
Legends for movies S1 to S14
Legend for table S1

Other Supplementary Material for this manuscript includes the following:

Movies S1 to S14
Table S1

Supplement Figures

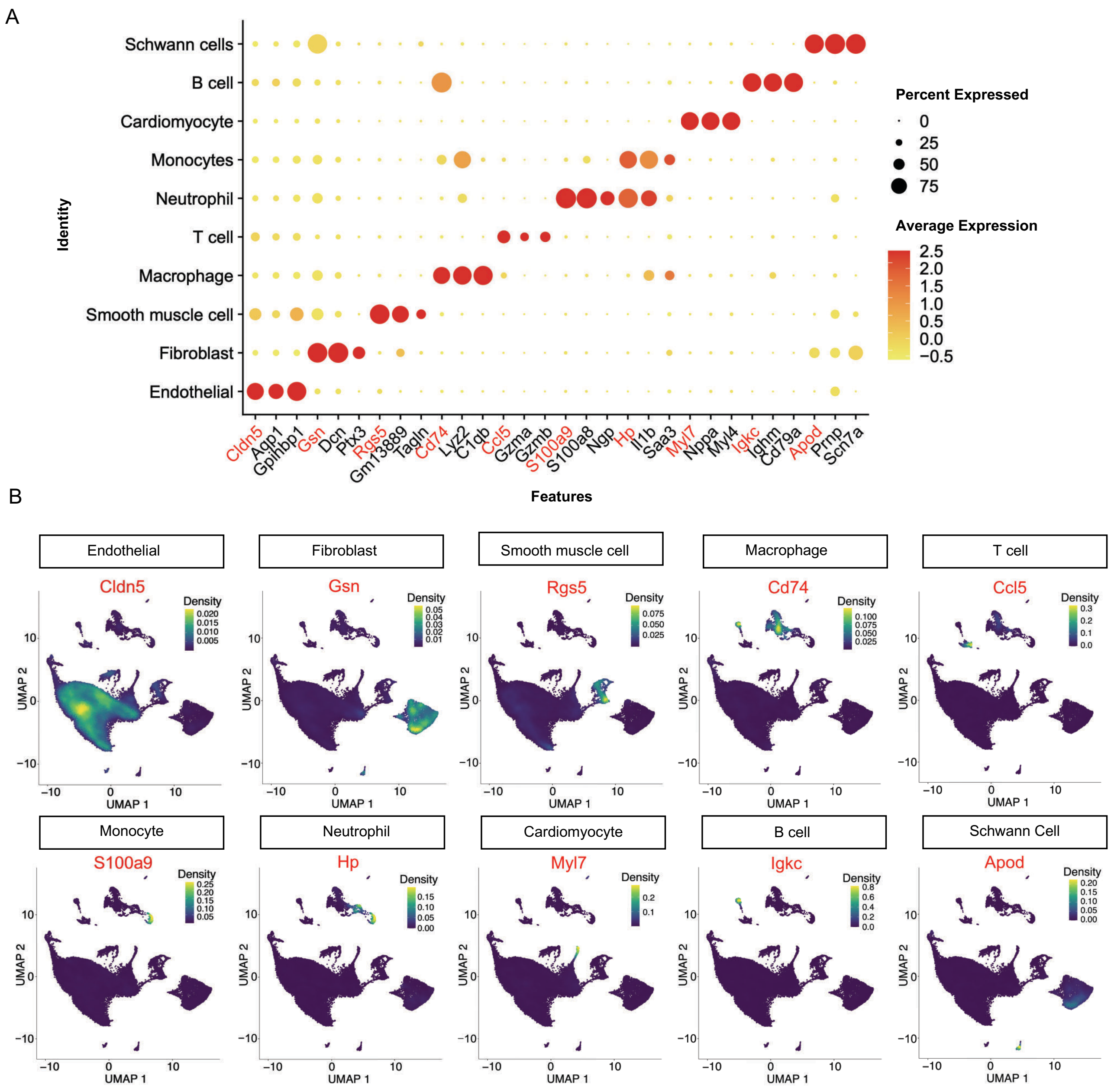

Supplementary Figure S1. Representative genes defining the 10 major cell clusters in septic heart tissue. (A) Dot plot of the average expression of the three marker genes in the 10 main cell clusters. **(B)** UMAP density map defining the genes for the 10 major cell clusters in each main cell type of the heart.

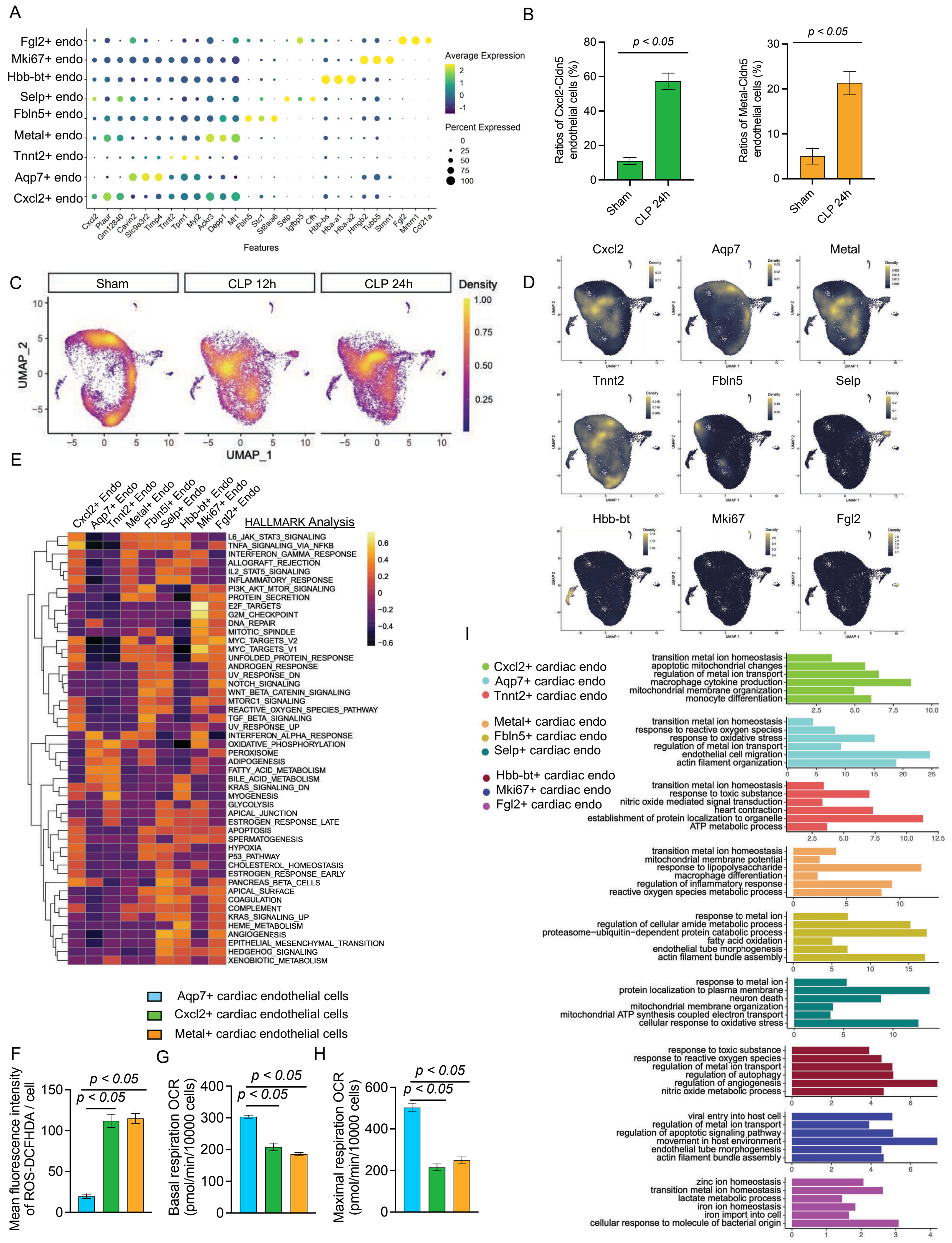

Supplementary Figure S2. Gene and functional analyses of each subcluster of cardiac endothelial cells. **(A)** Dot map of representative marker genes defining the nine groups of cardiac endothelial cell subclusters. **(B)**

Proportion of Cxcl2+ and Metal+ cardiac endothelial cells in different groups; n = 8 per group. **(C)** UMAP density plot of cardiac endothelial cell subclusters in the nine groups with increasing duration of sepsis. **(D)** UMAP density map defining representative marker gene expression and distribution of the nine groups of cardiac endothelial cell subclusters. **(E)** HALLMARKER functional analysis heatmap of nine groups of cardiac endothelial cell subclusters. **(F)** Mean fluorescence intensity of ROS in Aqp7+, Cxcl2+, and Metal+ cardiac endothelial cells; n = 3 per group. **(G-H)** Basal and maximal respiration analyses of Aqp7+, Cxcl2+, and Metal+ cardiac endothelial cells using flow cytometry; n = 3 per group. **(I)** GO enrichment analysis of functional changes in cardiac endothelial cell subclusters in the nine groups after sepsis. UMAP, uniform manifold approximation and projection; ROS, reactive oxygen species; GO, gene ontology.

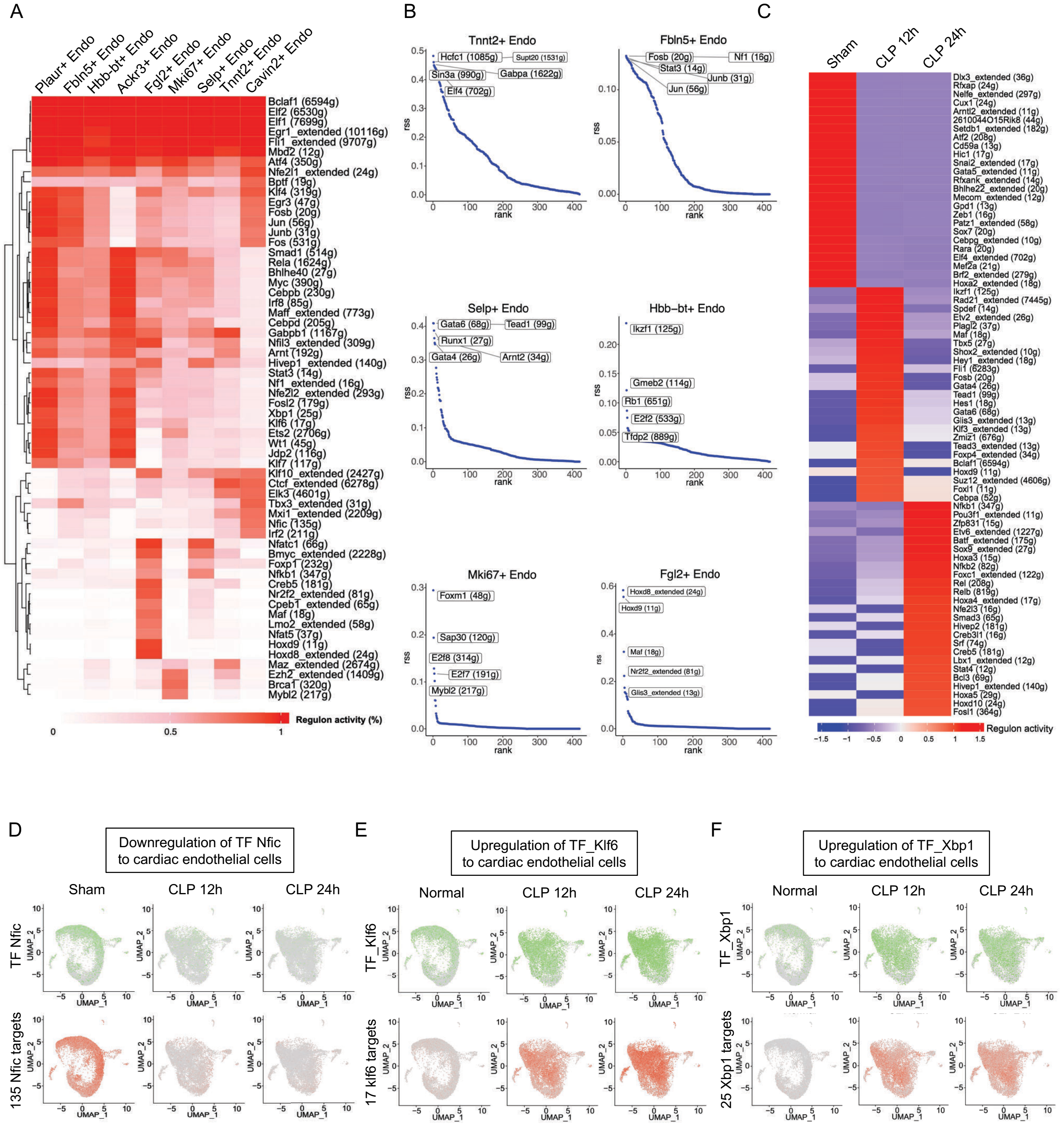

Supplementary Figure S3. Analysis of TFs in subclusters of cardiac endothelial cells. (A) AUC score heatmap of TFs expression regulation in each subcluster of cardiac endothelial cells, estimated using landscape analysis. **(B)** Top five TFs with the largest differences in the estimated expression regulation of the Tnnt2+, Fbln5+, Selp+, Hbb-bt+, Miki67+, and Fgl2+ subclusters in cardiac endothelial cells after sepsis. **(C)** TFs with significant differences in expression estimates as the duration of sepsis increased. **(D)** Distribution of Nfic and its target genes that cause the downregulation of cardiac endothelial cell subclusters as the duration of sepsis increases. **(E)** Distribution of Klf6 and its target genes that lead to the upregulation of cardiac endothelial cell subclusters as the duration of sepsis increases. **(F)** Distribution of Xbp1 and its target genes that cause the upregulation of cardiac endothelial cell subclusters as the duration of sepsis increases. TF, transcription factor; AUC, area under the curve.

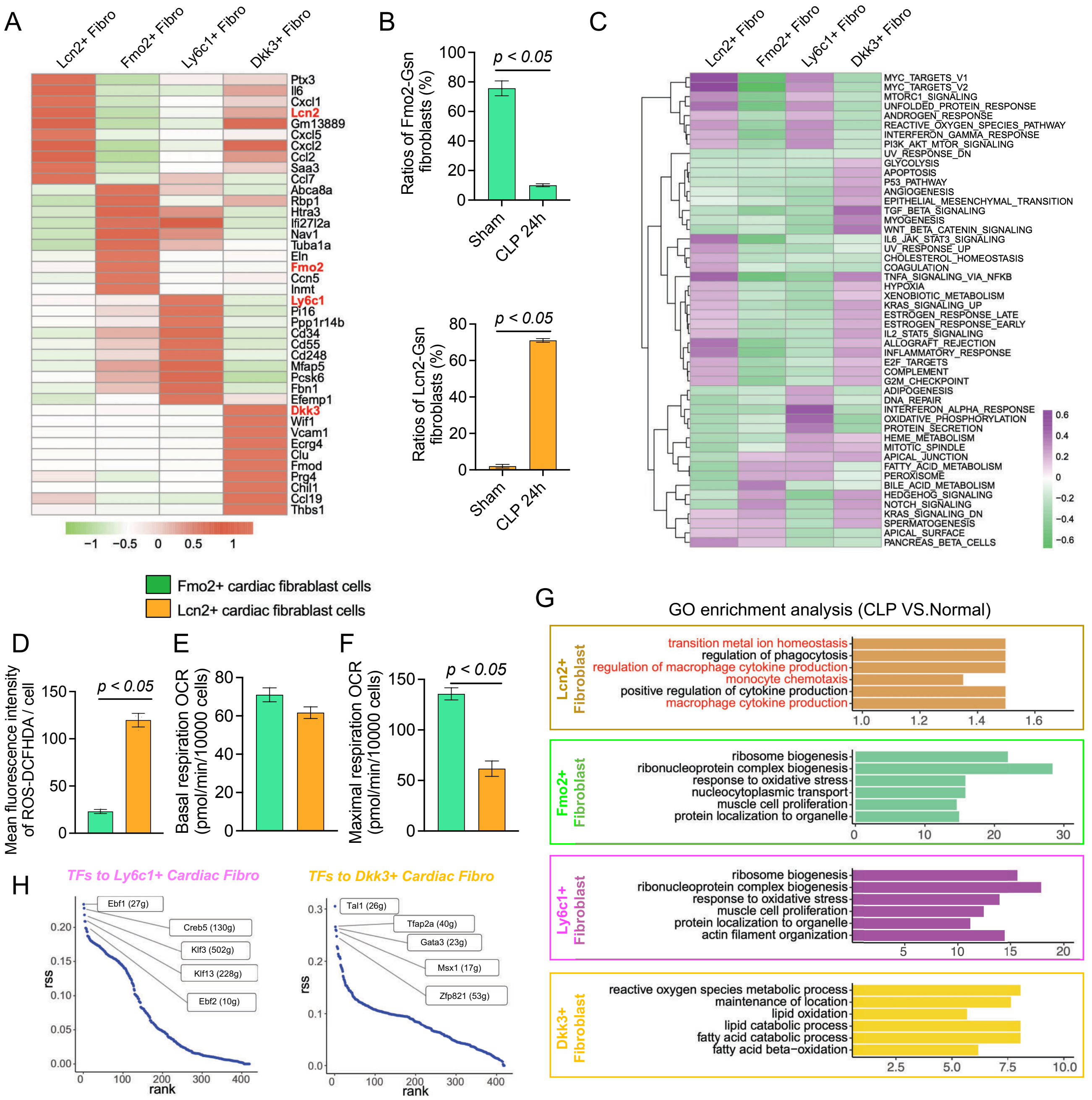

Supplementary Figure S4. Functional and TFs analyses of characteristic genes of each subcluster of CFs. (A) Heatmap of differentially expressed genes in the four subclusters of CFs after sepsis. **(B)** Proportion of Fmo2+ and Lcn2+ cardiac endothelial fibroblasts in different groups; n = 8 per group. **(C)** HALLMARK functional analysis heatmap of CF subclusters in the four groups. **(D)** Mean fluorescence intensity of ROS in Fmo2+ and Lcn2+ CFs as determined using flow cytometry; n = 3 per group. **(E-F)** Basal and maximal respiration analyses of Fmo2+ and Lcn2+ CFs after flow cytometry; n = 3 per group. **(G)** GO enrichment analysis of functional changes in CFs subclusters in the four groups after sepsis. **(H)** Top five TFs with the largest differences in regulatory expression estimates in the subcluster of CFs Ly6c1+ and Dkk3+ after sepsis. TF, transcription factor; ROS, reactive oxygen species; GO, Gene Ontology.

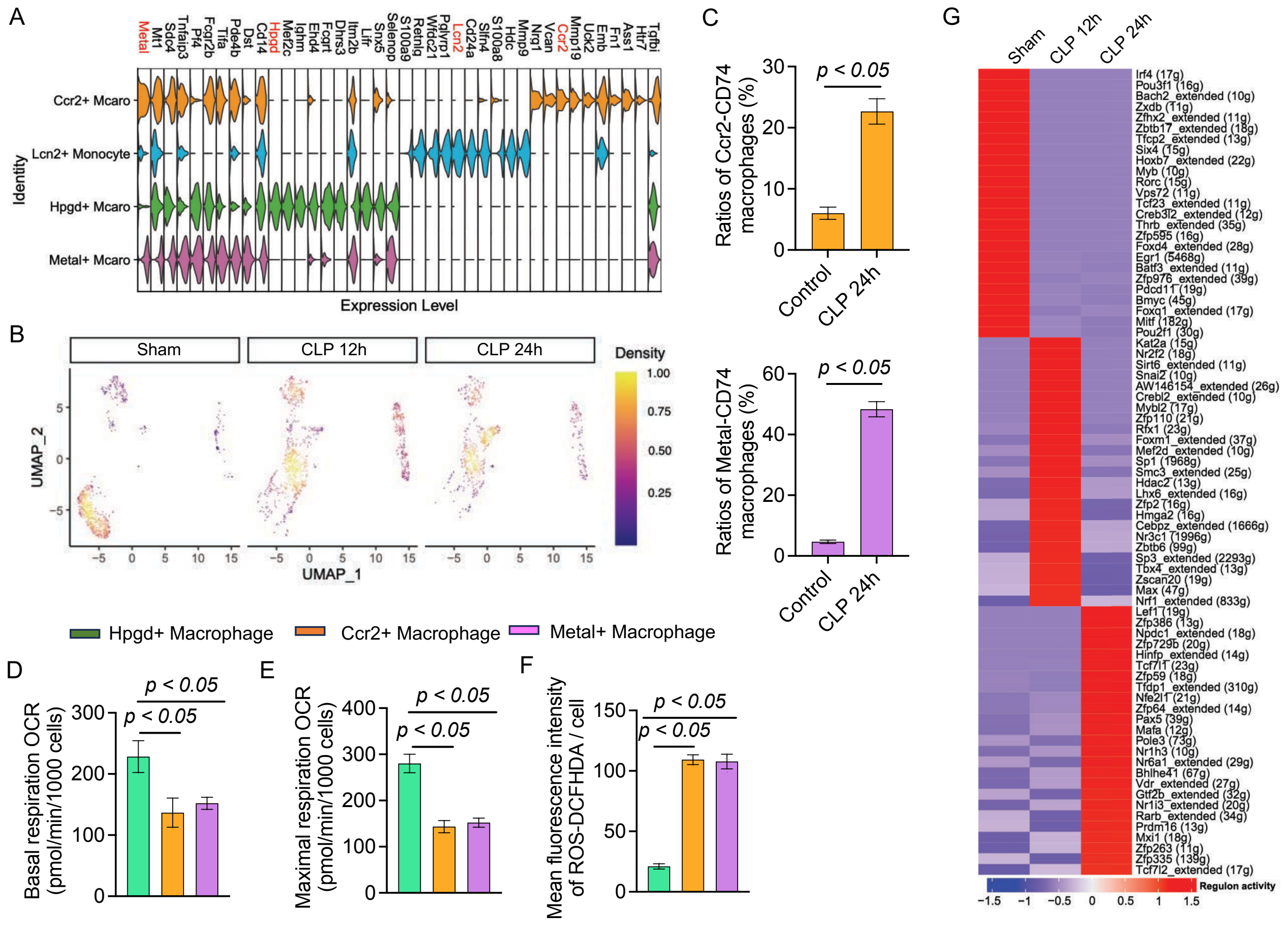

Supplementary Figure S5. Analysis of characteristic genes and TFs of each subcluster of cardiac monocytes-macrophages. (A) Violin diagram defining representative marker genes for the four groups of cardiac monocyte-macrophage subclusters. **(B)** UMAP density plot of cardiac monocyte-macrophage subclusters with increasing sepsis duration in the four groups. **(C)** Proportion of Ccr2+ and Metal+ cardiac macrophages in different groups; n = 8 per group. **(D-E)** Mean fluorescence intensity of ROS in Hpgd+, Ccr2+, and Metal+ cardiac macrophages after flow cytometry; n = 3 per group. **(F)** Basal and maximal respiration analyses of Hpgd+, Ccr2+, and Metal+ cardiac macrophages after flow cytometry; n = 3 per group. **(G)** TFs with significant differences in expression estimates as the duration of sepsis increased. TFs, transcription factor; UMAP, uniform manifold approximation and projection; ROS, reactive oxygen species.

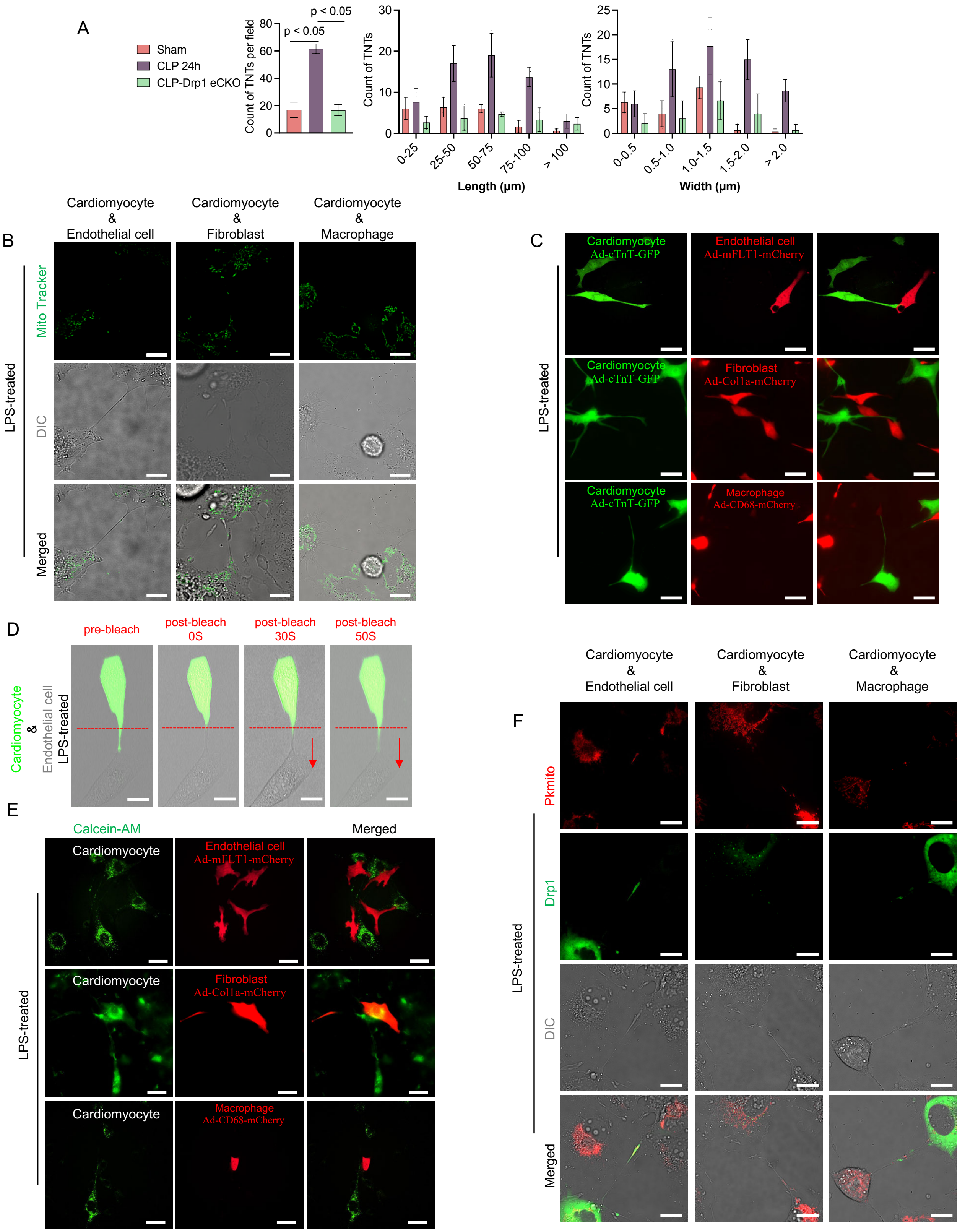

Supplementary Figure S6. (A) Bar graphs showing the number, length distribution, and width distribution of TNTs connecting cardiomyocytes with endothelial cells, fibroblasts, and macrophages, as calculated from the mIHC images in figure 5A; n = 6 per group. **(B)** Single-channel view of Figure 5B. Nanolive images showing the morphology of TNTs between primary cardiomyocytes and primary endothelial cells, fibroblasts, and

macrophages. Mitochondria were labeled with Mito Tracker (green); n = 3 per group; scale bars = 20 μm. **(C)** Immunofluorescence images showing TNT formation between cardiomyocytes (Ad-cTnT-GFP) and endothelial cells (Ad-mFLT1-mCherry), fibroblasts (Ad-Col1a-mCherry), and macrophages (Ad-CD68-mCherry); n=3 per group; scale bar = 25 μm. (**D**) FRAP analysis of TNTs between cardiomyocytes and endothelial cells. Red arrows indicate the direction of fluorescence recovery. **(E)** Immunofluorescence images showing TNT formation between cardiomyocytes (green, Calcein-AM) and endothelial cells, fibroblasts, and macrophages (red, mCherry) after co-culture; n=3 per group; scale bar = 25 μm. **(F)** Single-channel view of Figure 5F. HIS-SIM images showing the metastasis of mitochondria and Drp1 protein via TNTs between primary cardiomyocytes and primary endothelial cells, fibroblasts, and macrophages. Mitochondria were labeled with PKmito (red), Drp1 protein was labeled with Drp1 plasmids (green); n = 3 per group; scale bars = 10 μm. mIHC, multiplex immunohistochemistry; TNT, tunneling nanotube; HIS-SIM, structured illumination microscopy.

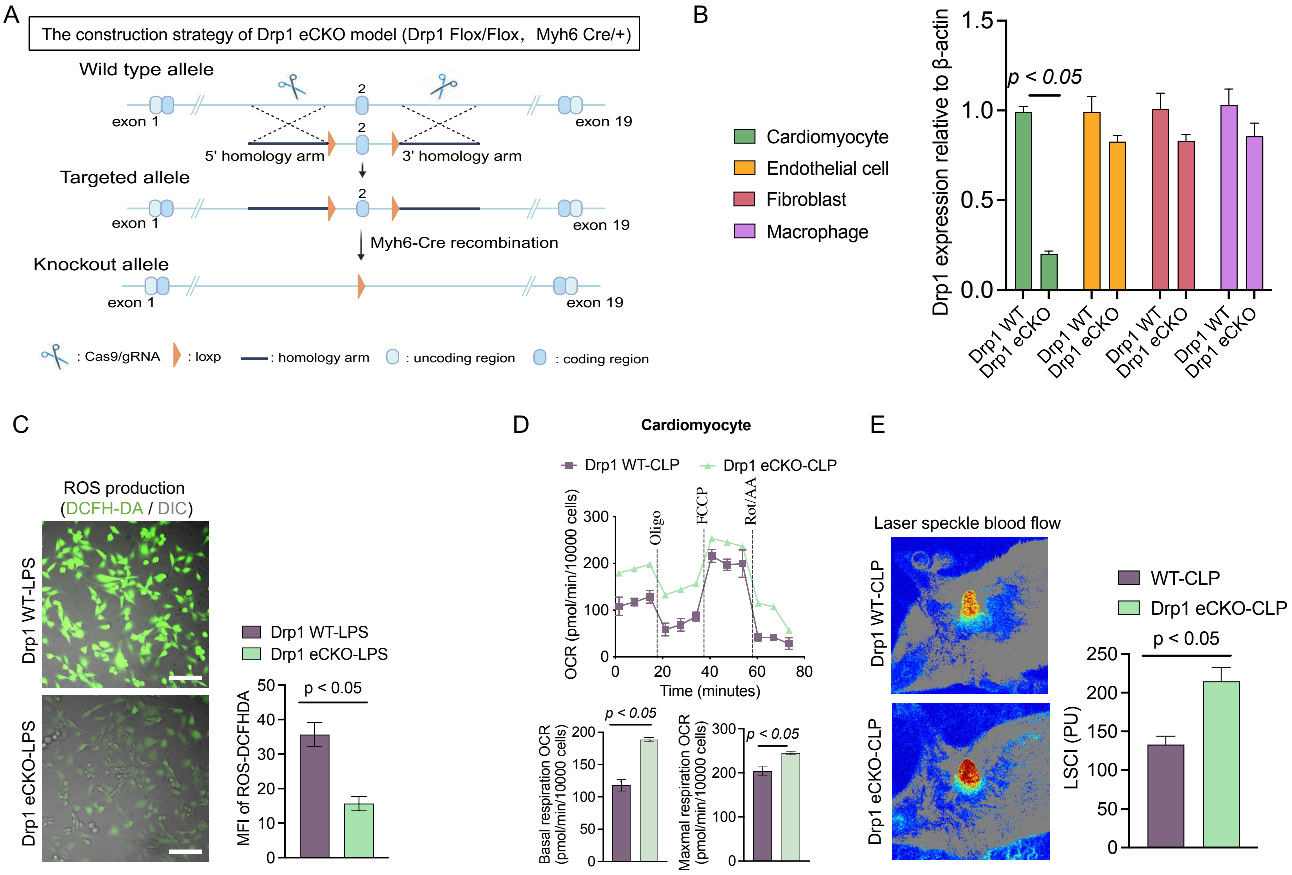

Supplementary Figure S7. (A) Scheme of the construction of cardiac-specific Drp1 conditional knockout mice. **(B)** RT-qPCR results confirmed Drp1 knockout efficiency in the cardiomyocytes, endothelial cells, fibroblasts, and macrophages of heart tissues; n = 8 per group. **(C)** ROS detection of primary cardiomyocytes; n=3 per group; scale bar = 50 μm. **(D)** Mitochondrial respiration capacity assays and basal and maximal respiratory profiling of primary cardiomyocytes from Drp1 WT-CLP and Drp1 eCKO-CLP mice; n=8 per group. **(E)** Laser speckle blood flow imaging and bar graphs showing cardiac perfusion from Drp1 WT-CLP and Drp1 eCKO-CLP mice; n=8 per group.

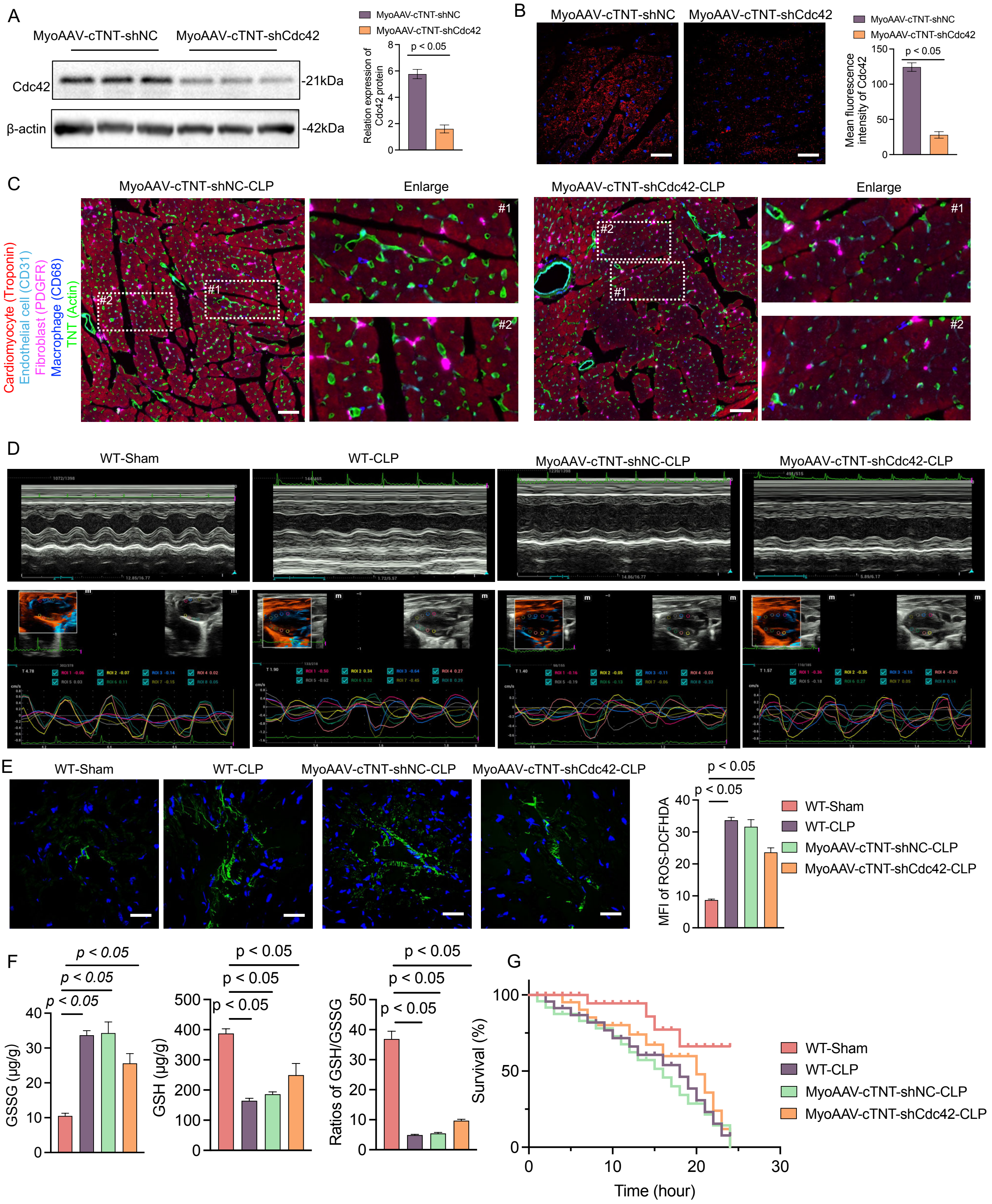

Supplementary Figure S8. (A) The protein expression of Cdc42 of heart tissue from MyoAAV-cTNT-shNC and MyoAAV-cTNT-shCdc42 mice, n=6 per group. **(B)** Immunofluorescence staining of Cdc42 expression in heart tissue from MyoAAV-cTnT-shNC and MyoAAV-cTnT-shCdc42 mice, n=6 per group. **(C)** mIHC staining of Cdc42 expression in heart tissue from MyoAAV-cTnT-shNC-CLP and MyoAAV-cTnT-shCdc42-CLP mice,

n=6 per group. **(D)** Ultrasound and endocardial/epicardial strain coefficients from WT-Sham, WT-CLP, MyoAAV-cTNT-shNC-CLP and MyoAAV-cTNT-shCdc42-CLP mice, n=6 per group. **(E)** ROS detection of heart tissue from WT-Sham, WT-CLP, MyoAAV-cTNT-shNC-CLP and MyoAAV-cTNT-shCdc42-CLP mice, n=6 per group; scale bar = 50 μm. **(F)** Bar graphs showing statistical analysis of GSSG, GSH contents and their ratio in cardiac tissues from WT-Sham, WT-CLP, MyoAAV-cTNT-shNC-CLP and MyoAAV-cTNT-shCdc42-CLP mice; n=6 per group. (**G**) Survival curves from WT-Sham, WT-CLP, MyoAAV-cTNT-shNC-CLP and MyoAAV-cTNT-shCdc42-CLP mice.

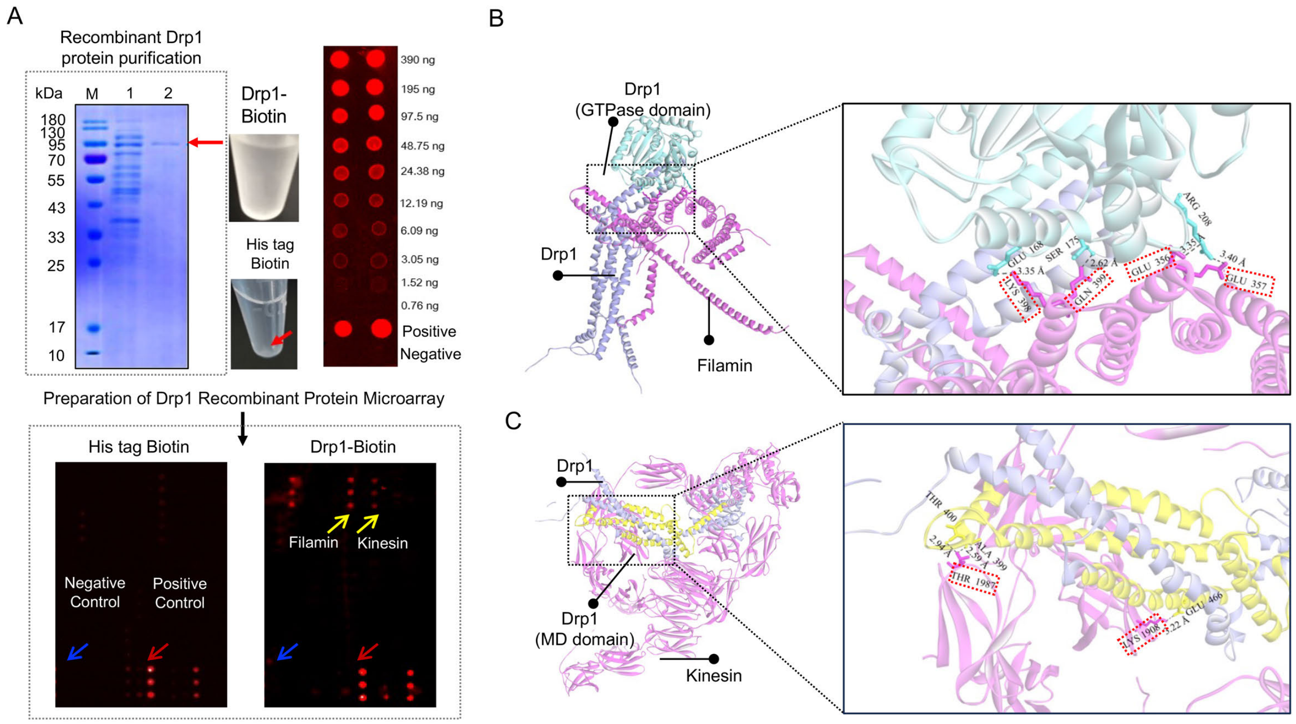

Supplementary Figure S9. (A) SDS-PAGE after purification of recombinant Drp1 protein (left). Concentration gradient plot of a standard sample in a chip (right). Samples and scanned images of the control group (6x His-tagged biotin) and experimental group (Drp1-biotin) on the chip, with red arrows indicating positive controls (biotin), blue arrows indicating negative controls (dimethyl sulfoxide), and yellow arrows indicating positive small-molecule screening results (below). **(B-C)** Schematic diagram of the docking of Filamin and Kinesin with Drp1.

Supplementary Videos

Supplementary Video 1. 3D scanning of TNT between cardiomyocyte and endothelial cell; scale bar = 10 μm. TNT, tunneling nanotube.

Supplementary Video 2. 3D scanning of TNT between cardiomyocyte and fibroblast cell; scale bar = 10 μm. TNT, tunneling nanotube.

Supplementary Video 3. 3D scanning of TNT between cardiomyocyte and macrophage; scale bar = 10 μm. TNT, tunneling nanotube.

Supplementary Video 4. 3D scanning of intercellular TNT with reconstruction using Imaris; scale bar = 4 μm. TNT, tunneling nanotube.

Supplementary Video 5. Time-lapse recording of mitochondrial metastasis via TNTs in primary cardiomyocytes and primary endothelial cells. Mitochondria were labeled with PKmito (purple); scale bar = 10 μm. TNT, tunneling nanotube.

Supplementary Video 6. Time-lapse recording of mitochondrial metastasis via TNTs between primary cardiomyocytes and primary fibroblasts. Mitochondria were labeled with PKmito (purple); scale bar = 10 μm. TNT, tunneling nanotube.

Supplementary Video 7. Time-lapse recording of mitochondrial metastasis via TNT between primary cardiomyocytes and the primary macrophages. Mitochondria were labeled with PKmito (purple); scale bar = 10 μm. TNT, tunneling nanotube.

Supplementary Video 8. Time-lapse recordings of the formation and extension of TNTs between Drp1 WT primary cardiomyocytes and primary fibroblasts. Mitochondria were labeled with PKmito (yellow); TNTs were labeled with actin (cyan); scale bar = 10 μm. TNT, tunneling nanotube.

Supplementary Video 9. Time-lapse recordings of the formation and extension of TNTs between Drp1 WT primary cardiomyocytes and primary endothelial cells. Mitochondria were labeled with PKmito (yellow); TNTs were labeled with tubulin (green); scale bar = 10 μm. TNT, tunneling nanotube.

Supplementary Video 10. Time-lapse recordings of the formation and extension of TNTs between Drp1 eCKO

primary cardiomyocytes and primary macrophages. Mitochondria were labeled with PKmito (yellow); TNTs were labeled with actin (cyan); scale bar = 10 μm. TNT, tunneling nanotube.

Supplementary Video 11. Time-lapse recordings of formation and extension of TNT between Drp1 eCKO primary cardiomyocytes and primary macrophages. Mitochondria were labeled with PKmito (yellow); TNTs were labeled with tubulin (green); scale bar = 10 μm. TNT, tunneling nanotube.

Supplementary Video 12. Time-lapse recordings of primary cardiomyocyte co-cultured with primary endothelial cells. Mitochondria were labeled with PKmito (purple); scale bar = 10 μm.

Supplementary Video 13. Time-lapse recordings of primary cardiomyocyte co-cultured with primary fibroblasts. Mitochondria were labeled with PKmito (red); scale bar = 10 μm.

Supplementary Video 14. Time-lapse recordings of primary cardiomyocyte co-cultured with primary macrophages. Mitochondria were labeled with PKmito (purple); scale bar = 10 μm.

Supplementary Table S1. Cell type markers of cardiac resident cells.
